# Hybrid Mass Spectrometry Applied across the Production of Antibody Biotherapeutics

**DOI:** 10.1021/jasms.4c00253

**Published:** 2024-11-22

**Authors:** Emilia Christofi, Mark O’Hanlon, Robin Curtis, Arghya Barman, Jeff Keen, Tibor Nagy, Perdita Barran

**Affiliations:** † Michael Barber Centre for Collaborative Mass Spectrometry, MBCCMS, Princess Street, Manchester M17DN, U.K.; ‡ Manchester Institute of Biotechnology, University of Manchester, Princess Street, Manchester M17DN, U.K.; § FUJIFILM Diosynth Biotechnologies, Belasis Ave, Stockton-on-Tees, Billingham TS23 1LH, U.K.

## Abstract

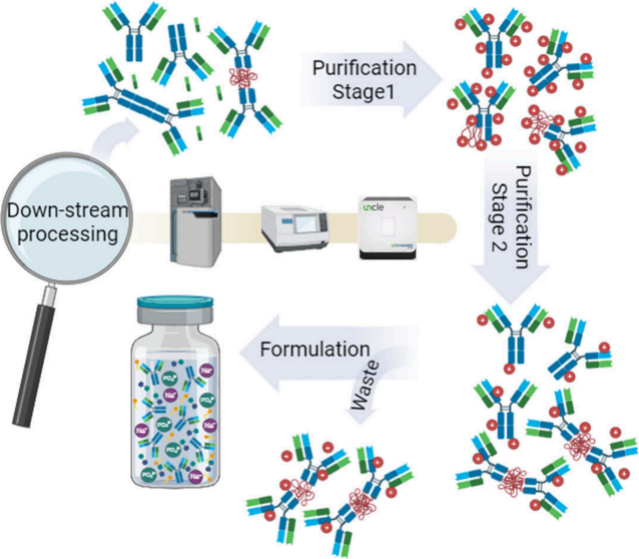

Post expression from the host cells, biotherapeutics undergo downstream processing steps before final formulation. Mass spectrometry and biophysical characterization methods are valuable for examining conformational and stoichiometric changes at these stages, although typically not used in biomanufacturing, where stability is assessed via bulk property studies. Here we apply hybrid MS methods to understand how solution condition changes impact the structural integrity of a biopharmaceutical across the processing pipeline. As an exemplar product, we use the model IgG1 antibody, mAb4. Flexibility, stability, aggregation propensity, and bulk properties are evaluated in relation to perfusion media, purification stages, and formulation solutions. Comparisons with Herceptin, an extensively studied IgG1 antibody, were conducted in a mass spectrometry-compatible solution. Despite presenting similar charge state distributions (CSD) in native MS, mAb4, and Herceptin show distinct unfolding patterns in activated ion mobility mass spectrometry (aIM-MS) and differential scanning fluorimetry (DSF). Herceptin’s greater structural stability and aggregation onset temperature (*T*_agg_) are attributed to heavier glycosylation and kappa-class light chains, unlike the lambda-class light chains in mAb4. Hydrogen–deuterium exchange mass spectrometry (HDX-MS) revealed that mAb4 undergoes substantial structural changes during purification, marked by high flexibility, low melting temperature (Tm), and prevalent repulsive protein–protein interactions but transitions to a compact and stable structure in high-salt and formulated environments. Notably, in formulation, the third constant domain (CH3) of the heavy chain retains flexibility and is a region of interest for aggregation. Future work could translate features of interest from comprehensive studies like this to targeted approaches that could be utilized early in the development stage to aid in decision-making regarding targeted mutations or to guide the design space of bioprocesses and formulation choices.

## Introduction

Monoclonal antibodies (mAbs) represent a pivotal advancement in biopharmaceuticals, distinguished by their specificity, selectivity, and biocompatibility.^1^ Their therapeutic efficacy extends to a broad spectrum of conditions, including various cancers^2−4^ and autoimmune disorders such as Crohn’s disease,^5,6^ rheumatoid arthritis,^7,8^ lupus,^9,10^ and more.^11,12^ The rapid expansion of the mAb market, projected to reach USD 445.6 million by 2028 according to Global Market Insights, underscores their significance in the field.^13^ The commercial development and manufacturing of mAb biotherapeutics are governed by rigorous standards established by regulatory bodies like the FDA and EMA.^14,15^ These standards, grounded in the Quality by Design (QbD) framework, focus on the impact of different variables on critical quality attributes (CQAs) including protein aggregates and glycosylation.^15−17^

Antibodies possess complex, dynamic structures that are crucial for their biological function. At elevated concentrations, however, they are prone to both reversible self-association and irreversible aggregation. With infusion concentrations ranging from 50 to 200 mg/mL, careful formulation is essential to mitigate these challenges.^18,19^ Aggregation poses significant issues throughout the production cycle—spanning expression, purification, formulation, and administration. Beyond reducing the effective concentration of the active pharmaceutical ingredient (API), aggregates can provoke adverse immune responses, escalate production costs, and lower product yield.^18,20^ Factors influencing aggregation include protein conformational and colloidal stability, which depend upon antibody primary sequence and structural properties (hydrophobic patches, surface charge anisotropy, conformational dynamics) as well as solution conditions (ionic strength, pH, excipient type and concentration, protein concentration).^18,21−32^

During early development, candidate antibodies are assessed for developability using predictive models and biophysical assays.^33−36^ Lead candidates subsequently undergo comprehensive biochemical and biophysical characterization.^37,38^ Hybrid mass spectrometry (MS) techniques, among other orthogonal methods,^27,29,39−42^ have been employed to examine the structural stability^43−46^ of mAbs with respect glycosylation,^15,17,44,47−51^ Hofmeister salts,^52−54^ high concentration,^55,56^ high agitation speed,^57^ heat stress,^58,59^ excipients^60^ and sequence mutations.^61,62^ Various chromatographic techniques, including reverse phase high-performance liquid chromatography (RP-HPLC), liquid chromatography–mass spectrometry (LC-MS), tandem liquid chromatography–mass spectrometry (LC-MS/MS), and size-exclusion chromatography coupled to mass spectrometry (SEC-UV-MS), are routinely employed to conduct forced degradation studies using a pH gradient. These studies are crucial for the development and formulation of biotherapeutics.^63−67^

This study presents a workflow to identify and assess structural changes in a model monoclonal antibody (mAb), termed mAb4, during downstream processing from perfusion media to the final formulated product. The workflow highlights key processing parameters that impact structural integrity, increase aggregation risk, and reduce the yield. Structural flexibility is assessed at the peptide level using hydrogen–deuterium exchange mass spectrometry (HDX-MS), while activated ion mobility mass spectrometry (aIM-MS) evaluates the structural stability of mAb4 under varying salt concentrations. Subsequently, a comparative gas-phase stability analysis of mAb4 and Herceptin, a well-characterized IgG1 antibody, was conducted. Sequence alignment revealed a sequence identity of 62% for the light chain and 92% for the heavy chain between mAb4 and Herceptin. This comparison aimed to evaluate the stability of the model mAb developed by FUJIFILM against a well-studied, commercially available antibody, demonstrating the value of integrated MS approaches.

To further understand how MS measurements inform on aggregation propensity, we also measure the diffusion interaction parameter (k_D_) through dynamic light scattering (DLS) and use thermal ramping to characterize the melting temperature (*T*_m_) and aggregation onset temperature (*T*_agg_) via differential scanning fluorimetry (DSF) and static light scattering (SLS). These parameters are commonly used to assess the colloidal stability of both native and unfolded states as well as the conformational stability of proteins. Although k_D_ and the second virial coefficient B_22_ (SLS) are often used interchangeably to gauge colloidal stability and reversible protein interactions, B_22_ reflects averaged protein–protein interactions through statistical mechanics, whereas k_D_ also incorporates hydrodynamic effects. Despite these distinctions, numerous studies demonstrate a strong, linear correlation between k_D_ and B_22_, underscoring their complementary roles in assessing protein interactions.^18,39,40,42^

We envisage that applying statistical methods to the multidimensional data generated by hybrid mass spectrometry (MS) approaches will facilitate the identification of key features, which can be translated into targeted strategies amendable with high-throughput screening (HTS). For instance, automated inlet systems for rapid charge state distribution assessments exemplify such targeted strategies.^68^ Assessing the critical features of interest and converting these to a rapid target could provide methods that are amenable to industrial scale up, and before this, insights into the effect of solution conditions from structural MS methods could be used to guide the design space for bioprocessing and formulation, improving the final product.

## Materials, Methods, and Analysis

**Figure 1:**
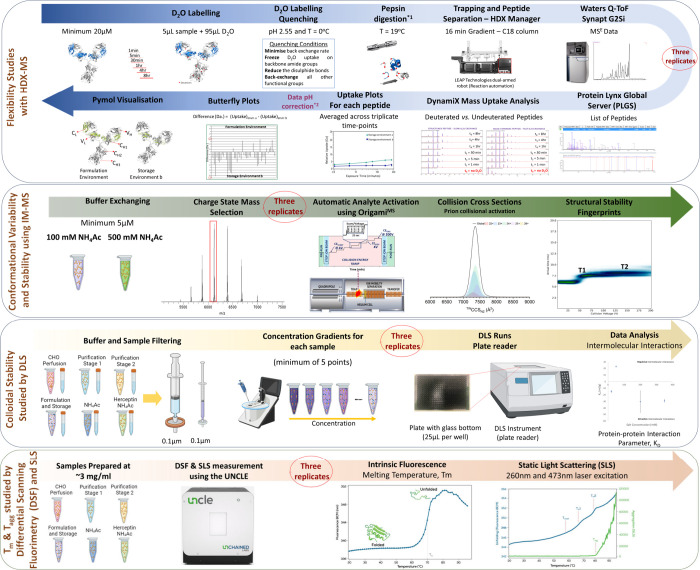
Hybrid MS and biophysical characterization. Experimental workflows adapted for the HDX-MS (blue arrow), aIM-MS (green arrow), DLS (yellow arrow), and DSF with SLS (orange arrow) (Table S4). For the HDX-MS experiments, the samples were diluted to 20 μM (2.938 mg/mL) using identical solution conditions to the samples. The mAb was labeled before being quenched, digested, and separated in a C18 analytical column. ProteinLynx Global server (PLGS), dynamX, HDflex, and Pymol were used for data processing, correction and visualization.^69^ IM-MS was used to assess the conformational variability of mAb4 and Herceptin under identical solution conditions. For the aIM-MS experiments, one of the samples was buffer exchanged in 200 mM and 500 mM NH_4_Ac and diluted down to 5 μM (0.735 mg/mL). Origami^MS^ was used for automated activation in the trap region. Origami^ANALYTE^ was used for data processing.^70^ For the DLS experiments, all six samples and solutions were filtered to remove any air bubbles or particulates that interfere with the readings. Concentration gradients were prepared using a minimum of five points. The data were then extracted and the interaction parameter, k_D_, was calculated. Lastly, for the DSF-SLS experiments, the samples were diluted to 3 mg/mL using the original solutions/buffers and run in triplicates using the UNCLE.^71^ The temperature was ramped from 20–95 °C using small increments of 1 °C. Intrinsic fluorescence measurements were performed to calculate the melting temperature, *T*_m_. SLS was incorporated in the experiments to measure the aggregation onset temperature, *T*_agg_, with the use of two lasers at wavelengths of 260 and 473 nm. Part of the workflow was created with BioRender.com. Part of the figure was adapted from ref (70). Copyright 2018 Elsevier. Reproduced with permission.

Our experimental approach, detailed in Figure 1 and Table S4, utilizes activated ion mobility mass spectrometry (aIM-MS) to probe mAb4’s structural stability across two salt concentrations. Structural flexibility is examined at the peptide level using hydrogen–deuterium exchange mass spectrometry (HDX-MS). The diffusion interaction parameter (k_D_), melting temperature (*T*_m_), and aggregation onset temperature (*T*_agg_) are evaluated through dynamic light scattering (DLS), differential scanning fluorimetry (DSF), and static light scattering (SLS), respectively. The workflow is applied to a model IgG1 mAb (mAb4) at four key stages of the downstream process.

### Materials

A model IgG1 monoclonal antibody (referred to as “mAb4”) was provided by FUJIFILM Diosynth Biotechnologies. The four sample points considered include perfusion media (PM), purification stage 1 (PS1), purification stage 2 (PS2), and the final formulated product (FP). These samples vary in ionic strength, pH, salt type, and excipients. Notably, PS1 presents an extremely acidic environment (pH ∼ 3.5) with low ionic strength, whereas PM, PS2, and FP are maintained within physiological pH ranges (5.5 < pH < 8.5), with PS2 exhibiting relatively high ionic strength (Table 1). Herceptin mAb was sourced from Roche-Genentech. IdeS (FabRICATOR ) was purchased from Genovis AB. NH_4_Ac, K_2_HPO_4_, KH_2_PO_4,_ Bradykinin, and formic acid were purchased from Fisher Scientific. Guanidine hydrochloride was purchased from Sigma-Aldrich. Purified water was produced in-house using a Milli-Q Advantage A10 system. LC-MS grade acetonitrile and acetic acid were purchased from VWR Chemicals. Polysorbate-20 was purchased from Cambridge Bioscience Limited. Na_2_HPO_4_, NaH_2_PO_4_, phosphine hydrochloride (TCEP), and Sucrose were purchased from Fluorochem Limited. Micro–Zeba Spin 7K MWCO desalting columns were purchased from Thermo Scientific. Purified water was produced in-house using a Milli-Q Advantage A10 system. Thin-wall filament glass capillaries with the dimensions 10 cm length × O.D 1.2 mm × I.D 0.69 mm were purchased from World Precision Instruments, Stevenage, UK. Capillary tips were pulled in house using a P-200 Laser Micropipette puller from Sutter Instruments Co., Novato, CA, USA). Platinum wire for solution ionization was sourced from Goodfellow, Huntingdon, UK with a diameter of 0.125 mm.

**Table 1:** Storage Solution Compositions of the Downstream Sample Points Used for Our Study^a^

**Sample Points**	**Storage solution composition**	**pH**
**Purification Stage 1 (PS1)**	50 mM NaAc	3.5
**Modified Purification Stage 1 (PS1_MODIFIED_)**	200 mM NaAc	3.5
**Purification Stage 2 (PS2)**	350 mM NaAc	6.0
**Formulation Product (FP)**	20 mM Na-Phosphate, 7.5% w/v Sucrose, 0.01% w/v Polysorbate 20	6.0
**mAb4 - buffer exchanged**	200 mM NH_4_Ac	6.8
**Herceptin - buffer exchanged**	200 mM NH_4_Ac	6.8

a Both mAb4 and Herceptin were also buffer exchanged in NH_4_Ac with identical solution composition. To assess any residual self-association in solution, mAb4 was prepared at four times the ionic strength of PS1 while maintaining a low pH.

### Methods and Analysis

Our experimental approach is detailed in Figure 1 and Table S4. For direct infusion experiments, approximately 5–10 μL of sample was loaded into thin-walled filament capillary tips. All direct infusion samples were buffer-exchanged in 200 mM NH_4_Ac, pH 6.8 at 0.735 mg/mL^–1^ (∼5 μM). The samples were ionised by applying positive potential (0.8–1.2 kV) to the solution through a platinum wire passing through the capillary tip.

### Collision-Activated Ion Mobility Mass Spectrometry, IM-MS

Activation experiments were performed on a Waters Synapt G2 using nano-ESI and trap-activated ion mobility; capillary voltage 0.85–1 kV, cone voltage 10 V, extraction cone voltage of 0.1 V, and source temperature of 30 °C. A higher backing pressure of ∼5.68 mbar was used to enhance desolvation and the trap cell was pressurized at 3.57 e^–2^ mbar with argon gas. For mobility separation, the ion mobility cell was operated at 8.9 mbar (40 V wave height and 600 m s^–1^ wave velocity). ORIGAMI^MS^ application from the Origami^70^ software was used for the automated acquisition of the activation data using a trap collision energy range of 0–200 V, an increment step of 2 V, and 6 scans (5 s each) per voltage step. ORIGAMI^ANALYTE^ was used for data processing. Herceptin mAb sample was prepared in 200 mM NH_4_Ac and mAb4 was prepared in both 200 and 500 mM NH_4_Ac, all at 5 μM and pH 6.8. Triplicate runs under identical conditions were performed to ensure data reproducibility. The salt concentration for aIM-MS experiments reflects real-world production conditions, where the antibody encounters varying ionic strengths.

### Hydrogen–Deuterium Exchange Mass Spectrometry, HDX-MS

HDX-MS tracks isotopic exchange of backbone amide hydrogens to map solvent-accessible sites and probe protein structure and dynamics.^72−74^ Ionic strength and pH significantly affect intrinsic exchange rates.^24,72,73,75−78^ The Lindestrøm-Lang model, which assumes protein “breathing” motions under native conditions, becomes unreliable outside physiological conditions,^73^ particularly at low pH (<5.5), where exchange occurs via an unfolding pathway. Woodward’s two-process model,^79,80^ later applied by Finucane et al.,^78^ accounts for these pathways. To normalize data across varying pH and ionic strengths, we calibrated intrinsic exchange rates using the unstructured Bradykinin peptide as a control, following the method of Seetaloo et al.^69^ Bradykinin and mAb4 samples were prepared under identical conditions, with Bradykinin spiked into the perfusion medium (PM) sample due to the unavailability of PM without mAb4. The samples were diluted from the original stock down to 2.94 mg mL^–1^ (∼20 μM), followed by a 16-fold dilution during labeling/incubation and a further 13-fold dilution during quenching, resulting in an injection concentration of less than 0.073 mg/mL (0.5 μM).

The hybrid HDX-MS setup consisted of a Waters nano-Acquity UPLC system with ESI MS detection coupled to a LEAP Technologies dual-armed robot for sample preparation, incubation, quenching, and inlet injection. A Waters Synapt G2S mass spectrometer was operated in positive ion and resolution mode. The data acquisition was over a *m*/*z* range of 200–2500. For each sample, the labeling solution was prepared with identical ionic strength, salt type, and pH to avoid pH and ionic strength shifts upon mixing. The HDX reaction was quenched (1 M TCEP, 1.5 M guanidine hydrochloride, and 100 mM phosphate, pH 3.5) at 1 °C for 30 s before being injected into the HDX manager. The LC gradient flow rate was at 40 μL min^–1^ and the peptides were eluted over a 16 min gradient. The aqueous mobile phase contained Milli-Q water and 0.1% formic acid, and the organic mobile phase contained Acetonitrile and 0.1% formic acid. The online pepsin digestion was performed using a Waters Enzymate BEH Pepsin 2.1 mm × 30 mm column at 19 °C for 3 min before proceeding to the trap column for desalting and then to the analytical column (Waters Acquity UPLC BEH C18 1.7 μm, 1.0 mm × 10 mm) held at 0 °C in the HDX manager for chromatographic peptide elucidation. Each labeling time point was run in randomized technical triplicates to ensure the robustness and reproducibility of the method.

Waters MassLynx software v4.1 was used for data acquisition, and the LEAP dual-arm robot was controlled by a method developed in the HDX Director 1.0.3.9. Data processing analysis and correction were carried out with the Waters ProteinLynx Global Server 3.0.1, Waters DynamX 3.0 software, and HDflex.^69^ HDflex was used to correct/normalize the HDX data, using the formulated product (FP) data as the reference point. The MS proteomics data have been deposited to the ProteomeXchange Consortium via the PRIDE^81^ partner repository with the data set identifier PXD051563 and 10.6019/PXD051563. To allow access to the HDX data of this study, the HDX data summary Table S7 and the HDX data Tables S5 and S6 are included in the Supporting Information as per consensus guidelines.^82^

### Homology Model for mAb4

A homology model of the mAb4 antibody was generated utilizing the Antibody Modeler tool in MOE 2019.0102 software (Molecular Operating Environment (MOE), Chemical Computing Group ULC, 910–1010 Sherbrooke St. W., Montreal, QC H3A 2R7, Canada). The approach outlined by Maier and Labute^83^ was employed to conduct a homology search, with 4HK0 serving as the framework template for both antibody chains. For the complementarity-determining regions (CDRs), templates 4HK0, 6C5V, and 2DD8 were employed for the light chains CDRL1, CDRL2, and CDRL3, respectively. Similarly, templates 4HK0, 4HK0, and 4HG4 were used for the CDRs of the heavy chain CDRH1, CDRH2, and CDRH3, respectively. This chimeric template is 97% identical to the parent for the variable domain of the light chain (VL) region and 92% identical to the variable domain of the heavy chain (VH). Full length heavy and light chain sequences were provided as input, and Ig mode of the Antibody Modeler application was executed. In this mode, the variable domain (Fv) was constructed using the previously mentioned method, while the constant region (Fc) was modeled based on a solvated molecular dynamics (MD) snapshot derived from the 1HZH structure of IgG1.

### Dynamic Light Scattering, DLS

All of the solution and samples used were filtered using a 0.1 μM low protein binding Durapore filter unit by Millex – GV. A concentration gradient, with a minimum of 5 points, was prepared for each sample and the concentration was confirmed with the Thermo Scientific NanoDrop One. The samples were centrifuged for 2 min at 10 rpm to remove any microbubbles formed during pipetting. Triplicates, of 25 μL volume, were loaded on a plate, centrifuged for 2 min at 2500 rpm on an Eppendorf Centrifuge 5804 R, and placed into the DynaPro Plate Reader from Wyatt for diffusion coefficient measurements. The average hydrodynamic radius (*R*_*h*_) of the monoclonal antibody was calculated using the diffusion coefficient for each sample point, along with the standard deviations. The invert of the hydrodynamic radius was plotted as a function of the concentration gradient formed for each sample. The slope of the curve was then multiplied by the R of the antibody at infinite dilution (*R*_*h0*_) to calculate the interaction parameters, k_D_. Viscosity corrections were applied to *R*_*h0*__,_ as it is inversely proportional to viscosity.^84^

### Differential Scanning Fluorimetry (DSF) with Static Light Scattering (SLS) - UNCLE

All mAb4 (PM, PS1, PS2, FP, and NH_4_Ac) and herceptin (NH_4_Ac) samples were diluted to approximately 3 mg/mL using the original stock solutions prepared in-house. The UNCLE instrument (Unchained Laboratories, LLC), which integrates differential scanning fluorimetry (DSF), static light scattering (SLS), and dynamic light scattering (DLS), was employed for measuring the melting temperature (*T*_m_) and aggregation onset temperature (*T*_agg_). UNCLE utilizes a UV laser at 266 nm and a blue laser at 473 nm to excite intrinsic protein fluorescence during SLS. The intensity of SLS is dependent on the excitation wavelength and aggregate size, with the 266 nm laser being sensitive to smaller aggregates and the 473 nm laser to larger aggregates. Each well on the UNCLE cassette was loaded with 8.5 μL of sample, and the samples were incubated for 60 s prior to temperature ramping. The temperature was ramped from 20 to 95 °C using incremental steps of 1 °C, with a 60 s holding time at each temperature point. Triplicate runs for each sample were conducted, and the results were averaged for analysis. DSF was utilized to monitor changes in fluorescence corresponding to protein unfolding as the temperature increased. SLS was integrated to determine the *T*_*agg*_, which indicates the onset of aggregation when one of the antibody’s unfolding events occurs. The data were extracted from UNCLE software and analyzed using Excel.

## Results and Discussion

### What Is the Difference in the Structural Flexibility of the Model mAb4 across the Production Line Based on Its Solvent Accessibility?

**Figure 2:**
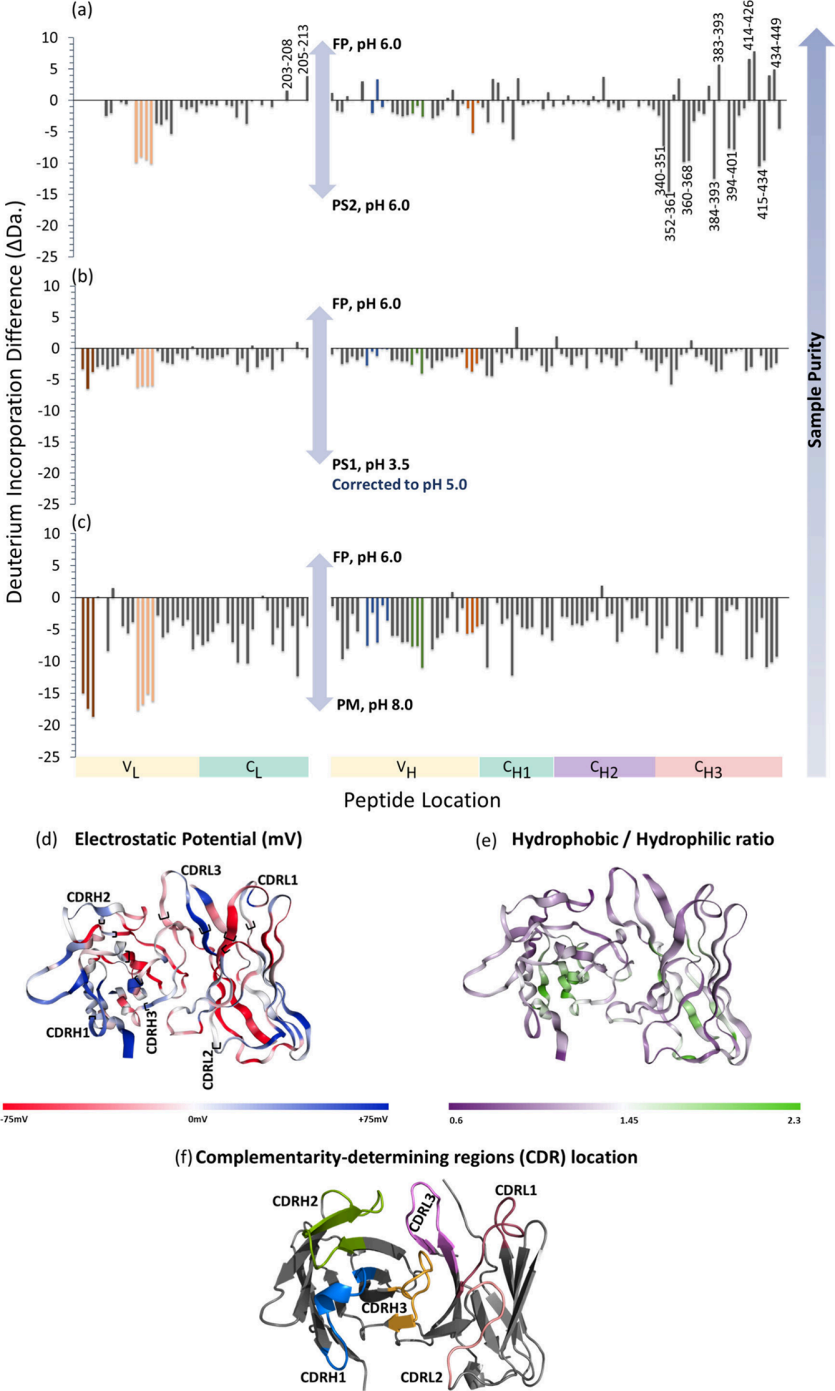
Deuterium incorporation difference plots for the light chain (LC) and heavy chain (HC) of mAb4. For ease of reference, the plots are displayed sequentially and are arranged from the N-terminus to the C-terminus, from left to right. The main subdomains of mAb 4: variable domains of LC and HC (VL, VH), and constant domains of LC and HC (CL, CH1, CH2, CH3) are labeled. Progressing from (c) to (a), the purity of the product increases. The interacting subdomains are shaded with the same colors (VL, VH = yellow) and (CL, CH1= green). The *y*-axis indicates the total D_2_O uptake difference, across all labeling times for (a) Purification step 2 (PS2) – Formulated Product (FP), (b) Purification step 1 (PS1) – Formulated Product (FP), and (c) Chinese Hamster Ovary (CHO) perfusion media (PM) – Formulated Product (FP). The location and morphology of the peptides containing the complementarity-determining regions (CDR) of the light chain, CDRL1 (5–26, 5–34,9–26), CDRL2 (42–76, 46–72, 49–72, 62–85), and the heavy chain, CDRH1, CDRH2, and CDRH3, are color-coded on the homology model of the mAb4 variable domain (f). The potential (d) and hydrophobicity (e) of the variable domain surfaces were assessed using the server at Protein-sol (https://protein-sol.manchester.ac.uk/patches).^96^ For the surface potential (d), blue is positive and red is negative, while for the polarity (e), purple is more polar and green is more nonpolar, with the CDR located between the brackets. The deuterium incorporation difference was calculated for each different peptide represented by the bars (HC sequence coverage = 96.2%, LC sequence coverage = 94.4%). The data for (b) were pH corrected down to 5 due to model limitations. The ionic strength of the solution increases with the following order Formulated Product *<* Purification Stage no.1 *<* Perfusion Media *<* Purification Stage no.2.

Using HDX-MS, we were able to monitor the flexibility (intramolecular interactions) of mAb4 across its production line (Figure 2). The FP is used as the reference state to allow comparison to other stages in the down-process. Formulated mAb4 exhibits reduced dynamics, especially the light chain (LC), where only two peptides (203–208 and 205–213) present significant deuterium uptake, and overall no uptake per peptide exceeds 5 Da over the 4 h time course (Figure 2a–c, Figure S4a). Under these formulation conditions the heavy chain (HC), also demonstrates reduced dynamics, although the third constant domain (CH3) has some dynamics, as shown by higher (>5 Da.) deuterium uptake for peptides spanning residues 383–393, 414–426, 434–449 (Figure S6). The FP solution environment contains a nonionic detergent, sucrose, as well as divalent anions (kosmotropes). The nonionic detergent although it does not directly stabilize the protein structure, it prevents interfacial adsorption which is the main cause of protein unfolding, hence reducing aggregation.^52,53,85,86^ Sucrose, which is a nonionic carbohydrates and is present in the formulation, enhances the conformational stabilization of mAb4 due to the preferential exclusion mechanism.^87,88^ These osmolytes are excluded from the hydration shell of the protein, decreasing the free energy of the system and driving the protein to a more compact state, due to the enhancement of the intramolecular interactions at the expense of protein–solvent interactions.^87,88^

Overall, mAb4 gains structural flexibility and increased solvent accessibility in the highly acidic elution solution of PS1 (Figure 2b,c). The solution pH plummets during the PS1 stage from pH ∼ 8 to pH ∼ 3.5, and some denaturation induced by this acidic environment is expected. Specifically, increased deuterium uptake can be seen in the VL (Figure 2a–c, Figure S4b–d), the VH (Figure S5a–c) and CH3 (Figure S6) of the HC, whereas the interface between the CH1 and CH2 domains seems to be preserved. The lower dynamics observed in the CH1/CH2 interface could be imparted by the nearby glycosylation site, N-297, which we have previously reported to stabilize antibody structure.^50^

We have also examined the protein’s behavior with a solution system commonly used in native MS experiments. The results in terms of deuterium uptake across the primary structure of HC and LC were qualitatively similar to those found in the PS2 solution, with lower uptake across both (Figure S1, Figure 2a). The ionic strength of this “native MS” solution is ∼1.8 times lower than that of PS2, and interestingly, here the mAb4 has a quantitatively lower deuterium incorporation across both LC and HC (Figure 2a, Figure S1). In the NH_4_Ac solution, peptides covering the CDRL2 (42–76, 46–72, 49–72, 62–85) and the CH3 (340–351, 352–361, 360–368, 384–393, 394–401, and 415–434), exhibit more than a 2-fold decrease compared to the PS2 (Figure S4, Figure S5b–d). The peptides at the CDRH1/2 interface show similar or lower magnitudes of deuterium incorporation in the PS2 solution compared with the NH_4_Ac solution (Figure S1, Figure 2a).

Similarly to the osmolytes found in the formulation solution, moderate to high concentrations (>100 mM),^89−91^ found in the PS2 and NH_4_Ac solution, are also known to enhance the structural stability of biomolecules.^52,85,86^ Kosmotropic ions are heavily excluded from around the protein increasing the surface free energy which is higher for the unfolded conformers (bigger surface area) compared to the folded conformers.^52,53,85,85,89,92,93^ This results in increase of the free energy of unfolding (*ΔGu*) and hence conformational stabilization.^52,53,85,85,89,92,93^ The differential experiments using NH_4_Ac (kosmotrope) provide good evidence of its ability to preserve a native form, as required in direct infusion MS. Our data show that under these conditions the protein dynamics are dampened compared with the solution(s) typically employed in expression and purification during manufacturing.

Another noteworthy observation is that the peptides containing the CDRL1 (5–26, 5–34, 9–26) and CDRL2 (42–76, 46–72, 49–72, 62–85) of the VL domain display some of the highest deuterium uptake in samples taken throughout the production as well as in the MS-compatible solution (Figure 2a–c, Figure S1, Figure S4b–d). The peptides containing the CDRs of the LC have higher deuterium uptake than those in the HC (Figure S7), suggestive of more dynamics. The entire variable region of mAb4 containing the CDRs is hydrophilic (Figure 2d,e) and the HDX data show the extent of this. With the use of a homology model, it can be seen that all three CDRs possess small secondary structural elements as well as several looped regions (Figure 2f). CDRL1 (5–26, 5–34,9–26) and CDRL2 (42–76, 46–72, 49–72, 62–85) primarily map to loops and extended regions, in line with our observation of higher deuterium uptake (Figure 2a–c, Figure S4). The LCs of mAb4 are of the lambda class and can contribute to the higher flexibility of its CDRs compared to those on the HC of the antibody. Townsender et al. and Honegger et al. report that lambda and kappa class LC exhibit different physiochemical and structural differences.^94,95^ Structurally, variable domains with lambda-class LC have lower thermodynamic stability, lower pI on average, and lower yield of protein expression in soluble form compared to the kappa-class LC.^95^ The CDR of the lambda-class LC, are mainly composed of different length loops compared to kappa-class LC,^94^ which along with the homology model is in support of our experimental results.

### Structural Organization Trends in Higher Salt Concentrations via Native MS and Activated IM-MS

**Figure 3:**
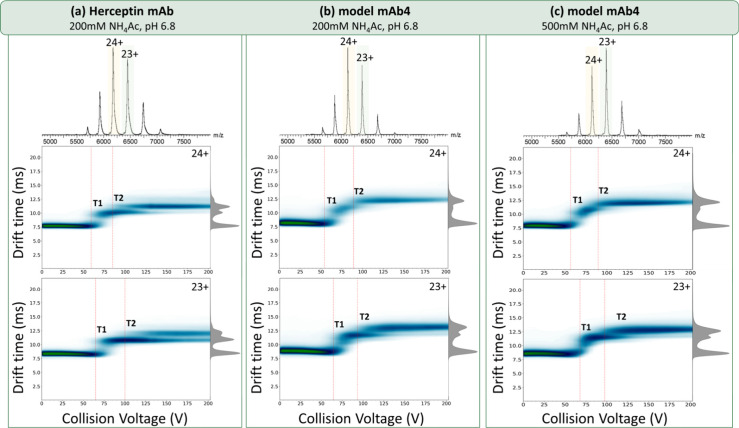
Native mass spectrometry data and activated IM-MS data for Herceptin (a), FUJIFILM Diosynth Biotechnologies mAb4 in 200 mM NH_4_Ac (b) and 500 mM NH_4_Ac (c). From the native mass spectra, a shift to lower charge states is observed at higher salt concentrations (c), while the width of the charge state distribution (CSD) remains the same. Both IgG1 mAbs present similar CSD (a,b) under similar solution conditions. Herceptin mAb undergoes two conformational transitions (T1 and T2) similar to mab4, but has slightly earlier drift times, even prior to activation. mAb4 shows favoring of conformers with higher structural stability at higher salt concentrations (+23). Before activation, mAb4 in both low and high ionic strengths (b,c) exhibits highly similar drift times, with that in high salt content being slightly lower, an indication of a slightly more compact initial conformation.

To assess the conformational stability of the FUJIFILM Diosynth Biotechnologies model mAb4 in both low and high salt concentrations, we employ native MS approaches (Figure 3). At the intact protein level, native MS readily reports on the role played by the ionic strength of the solution on the conformational heterogeneity of mAb4 (Figure 3). The charge state distribution (CSD) of mAb4 is highly similar to that of Herceptin, both centered on +23 and +24, when sprayed from identical solvents, although they have different masses (148.1 kDa Herceptin, 146.9 kDa mAb4) and glycosylation profiles (Figure S10, Figure S11, Table S1, Table S2). At high salt concentrations, both mAb4 and Herceptin present a shift toward lower charge states, centered on +23 (Figure 3c, Figure S13), although the mass and glycosylation profile are identical to that shown at lower salt concentration (Figure 3b). This is indicative that higher salt concentration favors the presence of more compact conformations.

For each of the mAb charge states, we also obtained the ion mobility data. The drift time for +23 of Herceptin (200 mM NH_4_Ac) is 8.3 ms, for mAb4 at 200 mM NH_4_Ac is 8.8 ms and for mAb4 at 500 NH_4_Ac is 8.6 ms. Similarly, for the +24 of Herceptin the drift time is 7.8 ms, for mAb4 in 200 mM NH_4_Ac is 8.2 ms and for mAb4 at 500 mM NH_4_Ac is 7.9 ms. The calculated CCS values were approximately 73.3 and 74.5 nm^2^ for Herceptin and mAb4, respectively, at charge state 23+, and approximately 73.6 and 74.8 nm^2^ for Herceptin and mAb4, respectively, at charge state 24+ (Figure S14). Overall, Herceptin exhibits narrower CCS distributions compared to mAb4, although both occupy a similar CCS range (ΔCCS) (Figure S14 and Figure S15). To probe the relative stability of these conformers, we performed collisional activation prior to ion mobility, which can be output as a map of drift time vs activation voltage (lab frame) (Figure 3). Such heat maps are remarkably good at revealing the differences between mAbs (Figure 3a,b) and the effect of increased salt on mAb4 (Figure 3b,c). Considering +24 first, both Herceptin and mAb4 undergo two structural transitions (*T1*, *T2*), but at slightly different energies, (*T1*) occurs at 60 and 54 V for Herceptin and mAb4, respectively. In light of the observation above that Herceptin has a smaller CCS this suggests it also has more stabilizing interactions. Notably, for Herceptin, the conformation adopted after this first transition (*T1*) (DT, 10 ms) remains throughout further activation (61–200 V), and a fraction of ions undergo a second transition (*T2*) at around 85 V, (DT, 11.25 ms) (Figure 3a). In comparison, the conformation following the first transition (*T1*) for mAb4 in 200 mM NH_4_Ac (DT, 10.8 ms), undergoes a second transition (*T2*) at approximately 90 V to one with DT of 12.3 ms, and the smaller conformer (*T1*) is no longer observed at and above 100 V. The percentage change from the inactivated to the two activated conformers for Herceptin and mab4 is similar, but the occupancy and the relative stability of the activated forms is different.

For +23, a similar heat map was observed for both Herceptin and mAb4 with two major structural transitions (Figure 3). The conformer of Herceptin formed after the first transition (*T1*, DT of 11.7 ms) persists throughout the experiment, as for +24. In comparison, for mAb4 the conformer formed after *T1* is also persistent, although a larger proportion of the ions undergo a second transition (*T2*), with DT of 13 ms, by 125 V and only traces of the *T1* conformer are left by the end of the experiment (130–200 V).

Overall, under identical solution conditions and despite their sequence (and mass) differences, Herceptin appears to be structurally more stable and is more compact than mAb4. The enhanced stability may be partially attributed to heavier glycosylation in Herceptin^17,50^ (Figure S11), which we have previously shown to stabilize mAb fold,^17^ and possibly the thermodynamically more stable^95^ kappa-class LC chains found in Herceptin compared to the lambda-class LC chains found in mAb4. The structural compactness of Herceptin compared to the large and more flexible mAb4 is also evident from the Dynamic light scattering (DLS) where they present *Rh*_*o*_ of 5.38 and 5.61 nm, respectively. These data were obtained in native MS salt conditions (200 mM NH_4_Ac) and the value for Herceptin is in close agreement with literature values taken under different salt condtions.^97,98^

When sprayed from a higher ionic strength solution, mAb4 presents unfolding patterns almost identical to those when sprayed from the lower ionic strength solution (Figure 3b,c). The starting DT of all conformers in the higher ionic strength solution are slightly earlier (∼0.3 ms) and the activation voltages required to induce the transitions are higher by an average of 2 V. This suggests that the overall protein structure undergoes only minor changes, and that the higher ionic strength solution permits the maintenance of a stabilized conformer, even in the desolvated forms.

Comparison of the DT for each mAb and from different starting solutions as the activation voltage is increased shows how each has a significantly different profile and highlights the benefit of this experimental workflow to compare conformational trends. Based on the native MS data, it can be concluded that mAb4 maintains its structural stability and is slightly more compact in the solutions with a higher salt content. Higher salt concentration favors the presence of conformers with higher structural stability, evident by the shift to lower charge states during direct infusion experiments. This was also evidenced in the HDX experiments where the higher ionic strength dampens mAb4 dynamics (Figure 2a).

To optimize conditions for the aIM-MS experiments (Figure 3) we considered desolvation, by altering the voltages in the ESI source and established the importance of retaining native like noncovalent interactions at the expense of signal intensity as previously reported (Figure S9).^99^ As a rule of thumb, when the initial conformer is native-like, it first undergoes structural compaction prior to transition,^99^ which is not observed when the sample was ionised using harsher conditions (Figure S9b).

A critical factor when formulating a biologic is to consider its aggregation propensity, which can also be examined with MS approaches. Despite mAb4 adopting a slightly more extended conformation in contrast to Herceptin at 200 mM NH_4_Ac (Figure 3) it demonstrates considerable structural stability at the molecular level within the formulated product (Figure 2). This robust stability at the molecular level also translates into experiments at higher concentrations, revealing variations in the aggregation tendencies of the two monoclonal antibodies (Figure S9). These experiments indicate that when sprayed under identical conditions, Herceptin shows a greater susceptibility to aggregation compared to that of mAb4 (Figure S9). And considering the dynamic domains flagged in the HDX-MS experiments, it appears that the CH3 domain stands out as a particularly significant region of interest (Figure 2a, Figure S6). In general, determination of biotherapeutic stability is not exclusively reliant on individual factors such as pH, ionic strength, salt types, filtration techniques, purification stages, sequence, or intra- and intermolecular interactions. Instead, it is a delicate interplay and balance of these components, extending beyond the ones specified.

### Can Molecular-Level Insights into Intrinsic Stability Affect Molecular Associations?

**Figure 4:**
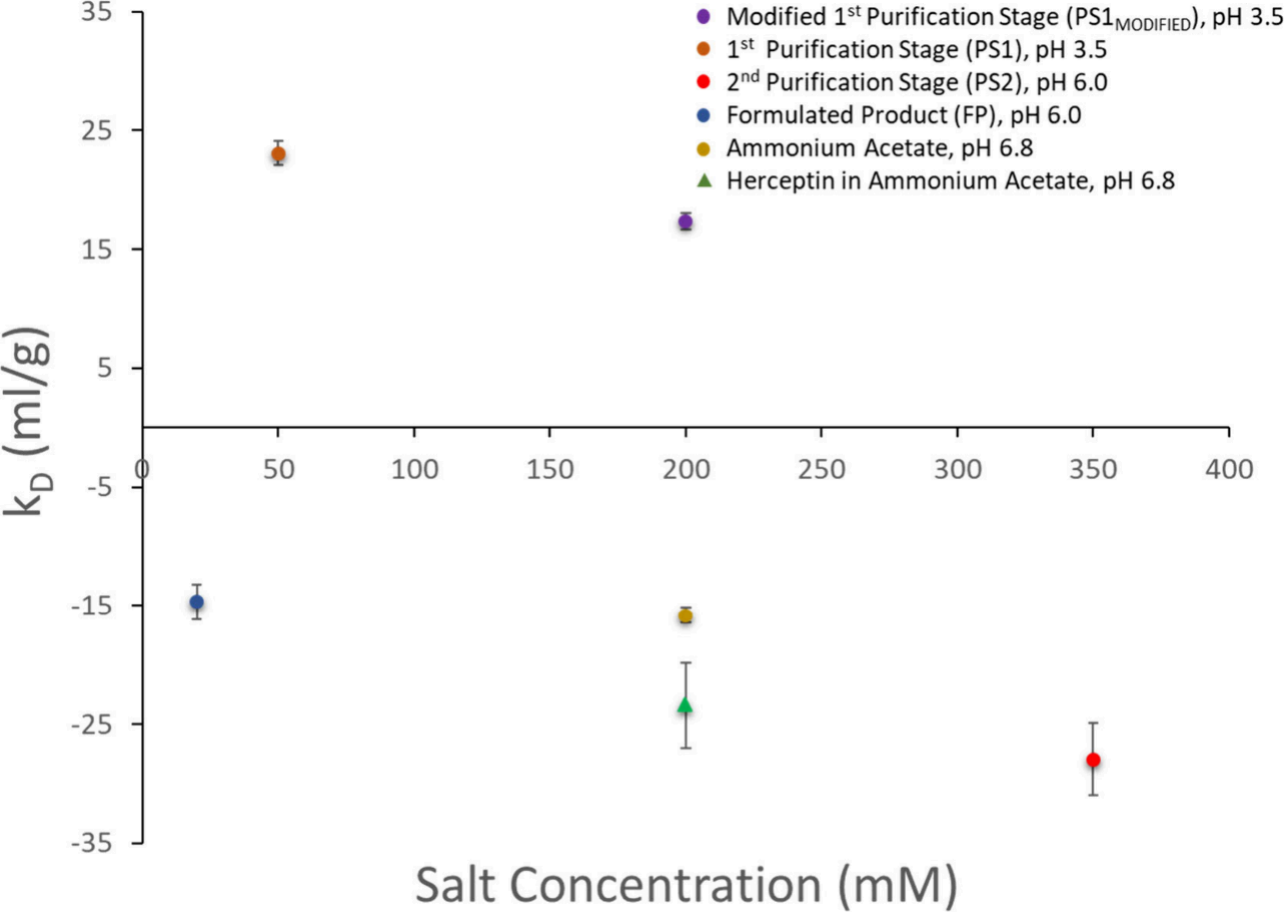
Diffusion interaction parameter (k_D_) calculated for mAb4 across the downstream process and for both mAb4 and Herceptin IgG1 prepared in an MS-compatible solution, with the use of dynamic light scattering (DLS). A negative value is indicative of attractive intermolecular interactions, and a positive value of repulsive intermolecular interactions in solution. Due to the presence of aggregates, resulting in high STDEV, an accurate k_D_ value could not be calculated for the perfusion media (PM) solution.

Dynamic light scattering (DLS) is commonly used to measure protein–protein interactions (PPI) or protein reversible self-association^100^ in terms of the diffusion interaction parameter (*k*_*D*_). This parameter is crucial for characterizing concentrated formulations, impacting properties such as viscosity, opalescence, and phase separation.^100,101^ The sample concentrations for the MS experiments are much lower than those typically used in formulation (>50 mg/mL),^102^ yet molecular-level data on structural stability should be able to inform on protein–protein associations occurring at higher concentrations. To bridge between the lower concentrations used for MS and those in the formulated product, we have measured *k*_*D*_ for each solution environment of interest (Figure 4, Table S3). Negative or positive *k*_*D*_ values are representative of attractive or repulsive intermolecular interactions, respectively.

In the highly acidic PS1 solution, strong repulsive intermolecular interactions dominate the behavior, making the determination of any remaining self-association interactions challenging. Measurements in solutions where the ionic strength of the PS1 solution is increased 4 fold, indicated no signs of strong self-association that might have been expected due to partial unfolding induced by the low pH (Figure 4, Table S3). In the elution solution of PS2, where the pH is within physiological range and the ionic strength is 7-fold greater than that of PS1 (Figure 4, Table S3) intermolecular interactions are much more attractive. Interestingly, similar levels of protein–protein attraction occur for the simple MS-compatible solution, which is at a comparable pH to the FP solution and almost 2-fold higher ionic strength (Figure 4, Table S3). In contrast, intermolecular interactions are more attractive for Herceptin compared to those of mAb4 (Table S3). The stronger attractive interactions observed for Herceptin could contribute to the heightened propensity for aggregation observed during native MS analysis compared to mAb4 (Figure S8).

Acidic environments are commonly used for elution during protein A chromatography and for viral particle inactivation. Solutions with pH much lower than the typical isoelectric point (pI) of ∼8.1 for mAb biotherapeutics cause a large net positive charge on the mAb. This, in turn, causes strong electrostatic repulsions between mAb monomers, which prevents aggregates from growing under stressed conditions (Figure 4).^78,103−106^ At a pH of 3.5, repulsive interactions also occur between domains within an antibody, causing localized unfolding and/or extensive flexibility of the tertiary structure, which can lead to increased hydrophobicity and reduced conformational stability. During neutralization from pH 3.5 (PS1) to pH 6.0 (PS2), the electrostatic repulsions between both irreversibly and reversibly unfolded monomers drastically reduce favoring the aggregation pathways. This is only true for partially folded states formed at low pH that do not refold quickly enough after adjusting to the higher pH of PS2.

This finding aligns well with observations from the HDX data, indicating that the dominant conformer in the monomeric state under the PS1 solution conditions is the most prone to aggregation across the entire production process, with the aggregation pathway most accessible as the mAb is taken from the PS1 to the PS2 conditions. According to the HDX-MS data, the CH3 (340–352, 352–368, 384–393, 394–401, 415–434, and 424–430), and CDRL2 (42–76, 46–72, 49–72, 62–85) display the biggest dynamicity and could potentially be two of the main contributors to the reversible or irreversible protein–protein interactions of mAb4 (Figure 2a,b).

Biotherapeutic stability manifests in various ways, with the balance between intra- and intermolecular interactions playing a crucial role in determining overall stability. Given that these therapeutics will eventually be administered directly to patients, it is imperative that their formulation solutions are compatible with human blood. This approach aims to mitigate toxicity, injection-related discomfort, and other potential adverse effects. Consequently, there are limitations in achieving complete stabilization of the final formulation, and necessary compromises or trade-offs need to be considered in this pursuit.

### Structural and Colloidal Stability of Mab4 across the Downstream Process

Differential scanning fluorimetry (DSF) combined with static light scattering (SLS) offers valuable insights into the conformational and colloidal stability of proteins during temperature ramping. This approach allows for simultaneous determination of the melting temperature (*T*_m_) and aggregation onset temperature (*T*_agg_), which provides insights into the relationship between unfolding and aggregation.

mAb4 exhibits similar primary melting temperatures (*T*_m1_) in PS2, FP, and NH_4_Ac solutions, with highly comparable *T*_agg_ closely aligned with *T*_m1_ values, indicating that aggregation is caused by the low temperature structural unfolding transition (Table 2, Figure S12). In contrast, mAb4 displays a notably lower *T*_m1_ and *T*_agg1_ of ∼39 °C in the PS1 solution, with fluorescence readings showing a distinct sharp increase even before heating, which is consistent with the enhanced flexibility observed at acidic conditions from HDX-MS and DLS experiments (Figure 2, Figure 4). Additionally, there is no detectable increase in 473 nm SLS signal, indicating only small aggregates occur and slow aggregate growth, which is consistent with the strong electrostatic repulsion occurring between proteins.

**Table 2:** Summary of the Average Melting Temperatures (*T*_m_) and Aggregation Onset Temperatures (*T*_agg_) for mAb4 across the Solution Environments Used in the Downstream Process as Well as for Both mAb4 and Herceptin in the MS-Compatible Solution^a^

		**DSF**		**SLS**		**aIM-MS (+23)**	
**Sample details**		***T*_*m1*_, (°C)**	***T*_*m2*_, (°C)**	***T*_*agg*_ (°C)**, 266 nm	***T*_*agg*_ (°C)**, 473 nm	***T1,* (V)**	***T2*, (V)**
**mAb4**	Purification Stage 1 (PS1)	39.07	69.92	39.86			
	Purification Stage 2 (PS2)	61.25	69.18	60.17	60.61	56^c^	90^c^
	Formulated Product (FP)	60.54	72.67	60.80	60.91		
	200 mM NH_4_Ac, pH 6.8	60.74	69.71	60.10	60.24	54	90
**Herceptin**	200 mM NH_4_Ac, pH 6.8	69.31	79.74	77.69	78.25	60^b^	100

a Voltages required for the structural transitions (T1 and T2) of the +23 charge state during aIM-MS are provided.

b The conformation adopted after this first transition (*T1*) remains throughout further activation (63–200 V), and a fraction of ions undergo a second transition (*T2*).

c The ionic strength of the PS2 is 350 mM ,whereas for activation a higher ionic strength of ∼500 mM was used.

Herceptin exhibits a *T*_m_ higher than that observed for any of the mAb4 samples. Notably, aggregation onset occurs near the second transition temperature, *T*_m2_, in contrast to mAb4 under identical solution conditions (Table 2). This greater thermal stability is likely attributed to increased glycosylation at N-297 in Herceptin (Figure S12) and the structural stability conferred by the kappa-class light chain compared to mAb4’s lambda-class light chain.^17,94,95,107,108^ The onset of aggregation occurring around T_m2_ suggests that there is a strong colloidal stability of the unfolded states formed in the low-temperature structural transition.

In summary, both DSF and aIM-MS studies strongly suggest two major structural transitions, supporting the use of MS methods to probe structural changes in bulk solution, with the advantage that they are directly probing the molecule of interest and are not influenced by aggregates. Our findings indicate Herceptin has higher structural stability compared with mAb4 as shown by ion activation prior to mobility studies and corroborated by higher values of *T*_m1_ and *T*_m2_ determined from the fluorescence readings.

## Conclusions

Our study demonstrates the utility of integrating mass spectrometry with fluorescence, dynamic light scattering (DLS), and static light scattering (SLS) to assess the structural stability, flexibility, and intermolecular interactions of biotherapeutics during downstream processing. We identified that mAb4 adopts an aggregation-prone conformation during early purification with aggregation further promoted by neutralization, as indicated by DLS. DX-MS revealed greater stability under higher ionic strength in the final purification stages, supported by a shift to more stable, lower charge states in aIM-MS and native MS. Increased deuteration in the light chain’s CDRs suggests higher flexibility compared to the VH region, likely due to hydrophilicity in CDRL1 and CDRL2, reflecting lambda-class and kappa-class dynamics. In contrast, Herceptin showed superior structural stability and aggregation onset only at higher temperatures, attributed to its heavier glycosylation and kappa-class light chain. Although higher salt concentrations lead to reduced molecular dynamics, the unchanged *T*_m_ values suggests that DSF alone may not fully capture stability at room temperature, warranting further investigation into the relationship between dynamics and aggregation.

Our integrated approach provides a framework for evaluating antibody stability and aggregation risks, informing targeted mutations, and optimizing formulation and purification processes. Future research should focus on applying statistical methods to extract key features from multidimensional MS data, enabling the development of targeted strategies for high-throughput screening (HTS). For example, automated systems for rapid charge state assessments could streamline this process. While immediate changes to purification protocols are unlikely, our findings offer valuable insights into antibody structural integrity across purification stages, supporting the optimization of bioprocess design. Over the next decade, incremental improvements in downstream processing are expected, as these insights are applied to enhance product stability and scalability, advancing the efficiency and efficacy of biopharmaceutical development.

## Data Availability

The mass spectrometry proteomics data have been deposited to the ProteomeXchange Consortium via the PRIDE59 partner repository with the data set identifier PXD051563 and 10.6019/PXD051563.

## Supporting Information Available

Additional figures and tables (PDF)
HDX data (XLSX)
HDX data summary (XLSX)
