# Supplementary material for: Hybrid Mass Spectrometry Applied across the Production of Antibody Biotherapeutics
Source: J Am Soc Mass Spectrom. 2024 Nov 22;36(1):44–57. doi: 10.1021/jasms.4c00253 (PMC11697328; doi:10.1021/jasms.4c00253)
Supplement: Supplementary file 1 — js4c00253_si_001.pdf [file js4c00253_si_001.pdf]

Supporting Information

Hybrid mass spectrometry applied across the production of antibody biotherapeutics.

Emilia Christofi[1,2], Mark O'Hanlon[2], Robin Curtis[2], Arghya Barman[3], Jeff Keen[3], Tibor Nagy[3], and Perdita Barran[1,2*]

[1] Michael Barber Centre for Collaborative Mass Spectrometry, MBCCMS, Princess Street, M17DN, UK

[2] Manchester Institute of Biotechnology, University of Manchester, Princess Street, M17DN, UK

[3] FUJIFILM Diosynth Biotechnologies, Belasis Ave, Stockton-on-Tees, Billingham, TS23 1LH, UK

* Address correspondence to perdita.barran@manchester.ac.uk

For Table of Contents Use Only

Figure S1: Deuterium Incorporation difference plots for the light chain (LC) and heavy chain (HC) of mAb4. 3

Figure S2: Coverage map of mAb4’ heavy chain. 3

Figure S3: Coverage map of mAb4’ light chain........ 3

Figure S4: Uptake plots generated using Hdflex for light chain peptides (a) presenting significant uptake in the formulation product, (b) covering the first complementarity determining region, CDRL1, (c) CDRL2, and (d) right after CDRL2........ 4

Figure S5: Uptake plots generated using HDflex for heavy chain peptides covering the (a) first complementarity determining region, CDRH1, (b) second, CDRH2, and (c) third CDRH3. 5

Figure S6: Uptake plots generated using HDflex for heavy chain peptides covering the third constant domain, CH3........ 6

Figure S7: Uptake plots generated using HDflex for heavy chain peptides (VH-CH2) presenting significant uptake in the formulation solution (FP). 7

Figure S9: Native mass spectrometry data and activated IM-MS data of mAb4 sprayed from 500mM NH_4Ac, pH 6.8 under soft ionisation parameters (a) and harsher ionisation parameters (b) 9

Figure S10: Deconvoluted mass spectrum associated with the glycosylated full-length mAb4 following native MS (a) and Fc/2 antibody subunit following incubation with IdeS (LC-MS). 10

Table S1: A summary of the LC-MS results for the digested mAb4. 11

Table S3: Interaction Parameters, kD, calculated using Dynamic Light Scattering (DLS) 13

Figure S12: Structural and Colloidal Stability studies using Differential Scanning Fluorimetry (DSF) coupled with Static Light Scattering (SLS). 14

Table S4: Volume and concentration requirements for the mass spectrometry and orthogonal techniques used for the experiments........ 15

Figure S13: Native mass spectrometry data for Herceptin in (a) 200mM NH_4Ac, and (b) in 500mM NH_4Ac........ 16

Table S5: Synapt G2Si (TWIMS) tuning parameters used for the IM-MS experiments........ 17

Figure S14: Collision cross section (CCS) distribution for (a) mAb4 and (b) Herceptin........ 18

Figure S15: Fitted global collision cross section (CCS) distributions for (a) mAb4, and (b) Herceptin…19

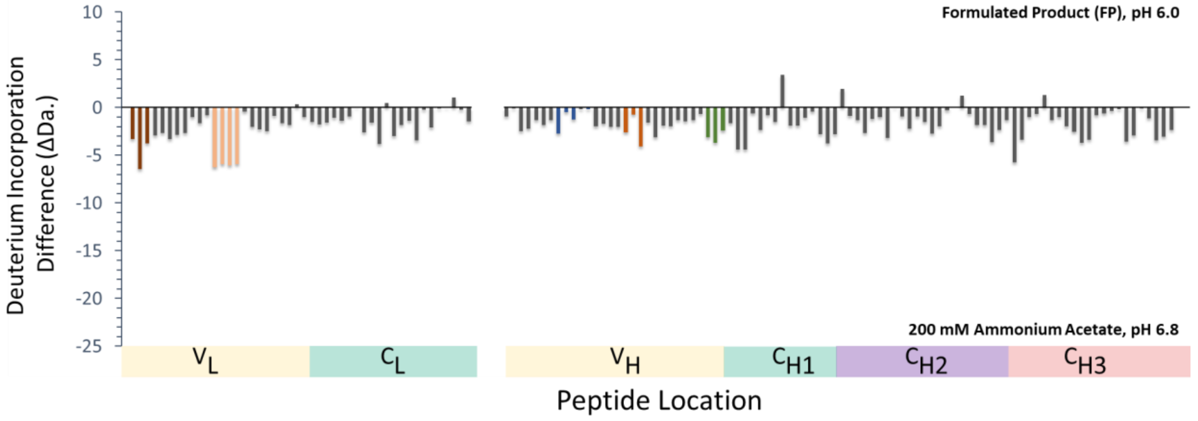

Figure S1: Deuterium Incorporation difference plots for the light chain (LC) and heavy chain (HC) of mAb4 (Formulation - NH_4Ac), which are shown sequentially in the figure for ease of reference. The main sub-domains of mAb 4: variable domains of LC and HC (VL, VH), and constant domains of LC and HC (CL, CH1, CH2, CH3) are labelled on the plots. The sub-domains interacting are shaded with the same colours (VL, VH – yellow) and (CL, CH1 – green). The y-axis indicates the total D_2O uptake difference, across all labelling times for Ammonium Acetate – Formulated Product (FP). The location and morphology of the peptides containing the complementarity-determining regions (CDR) of the light chain, CDRL1, CDRL2, and the heavy chain, CDRH1, CDRH2 and CDRH3 are colour-coded on the homology model of the mAb4 variable domain in Figure 2f.

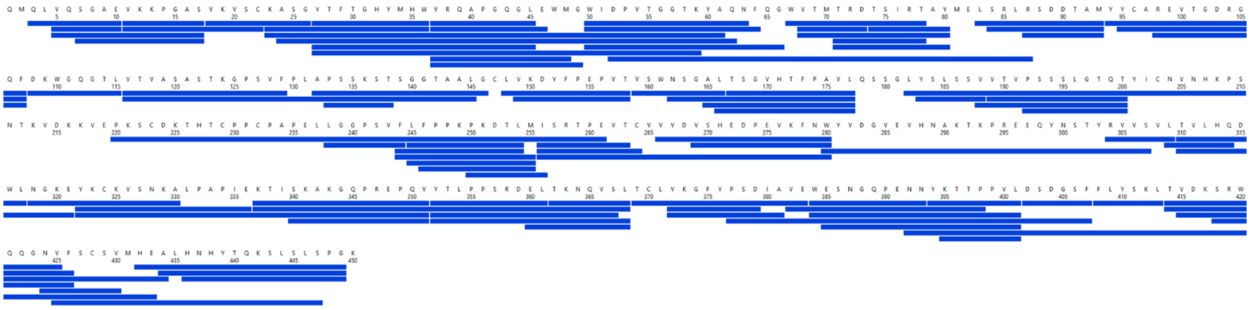

Figure S2: Coverage map of mAb4' heavy chain. After filtering, 98 peptides were chosen providing a 96.2% coverage and 3.3 redundancy. The minimum PLGS score chosen for the filtering was 7 and minimum product per amino acid of 0.2. Scores less than 7 resulted in extremely long peptides which were not insightful for our study.

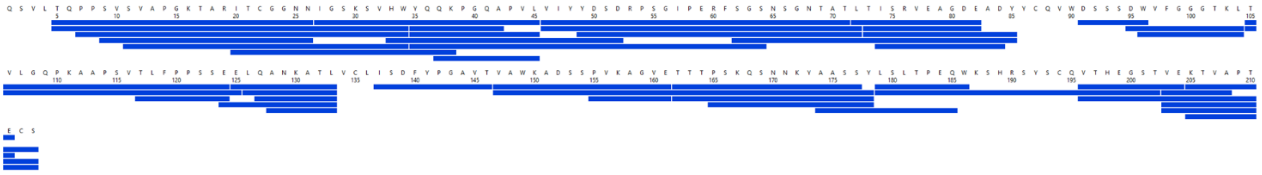

Figure S3: Coverage map of mAb4' light chain. After filtering 47 peptides were chosen providing a 94.4% coverage and 3.33 redundancy. The minimum PLGS score chosen for the filtering was 7 and minimum product per amino acid of 0.2. Scores less than 7 resulted in extremely long peptides which were not insightful for our study.

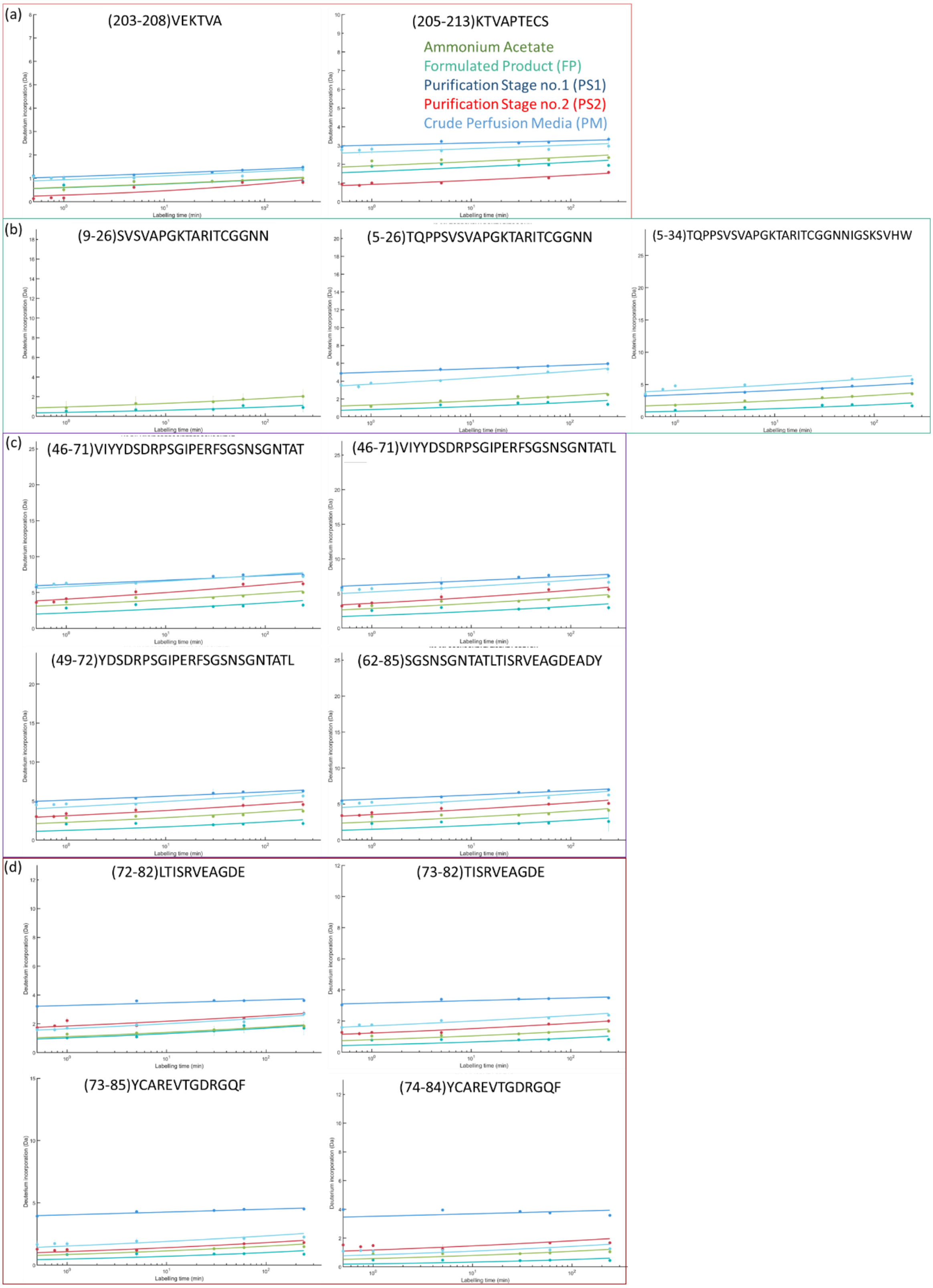

Figure S4: Uptake plots generated using Hdflex for light chain peptides (a) presenting significant uptake in the formulation product, (b) covering the first complementarity determining region, CDRL1, (c) CDRL2, and (d) right after CDRL2.

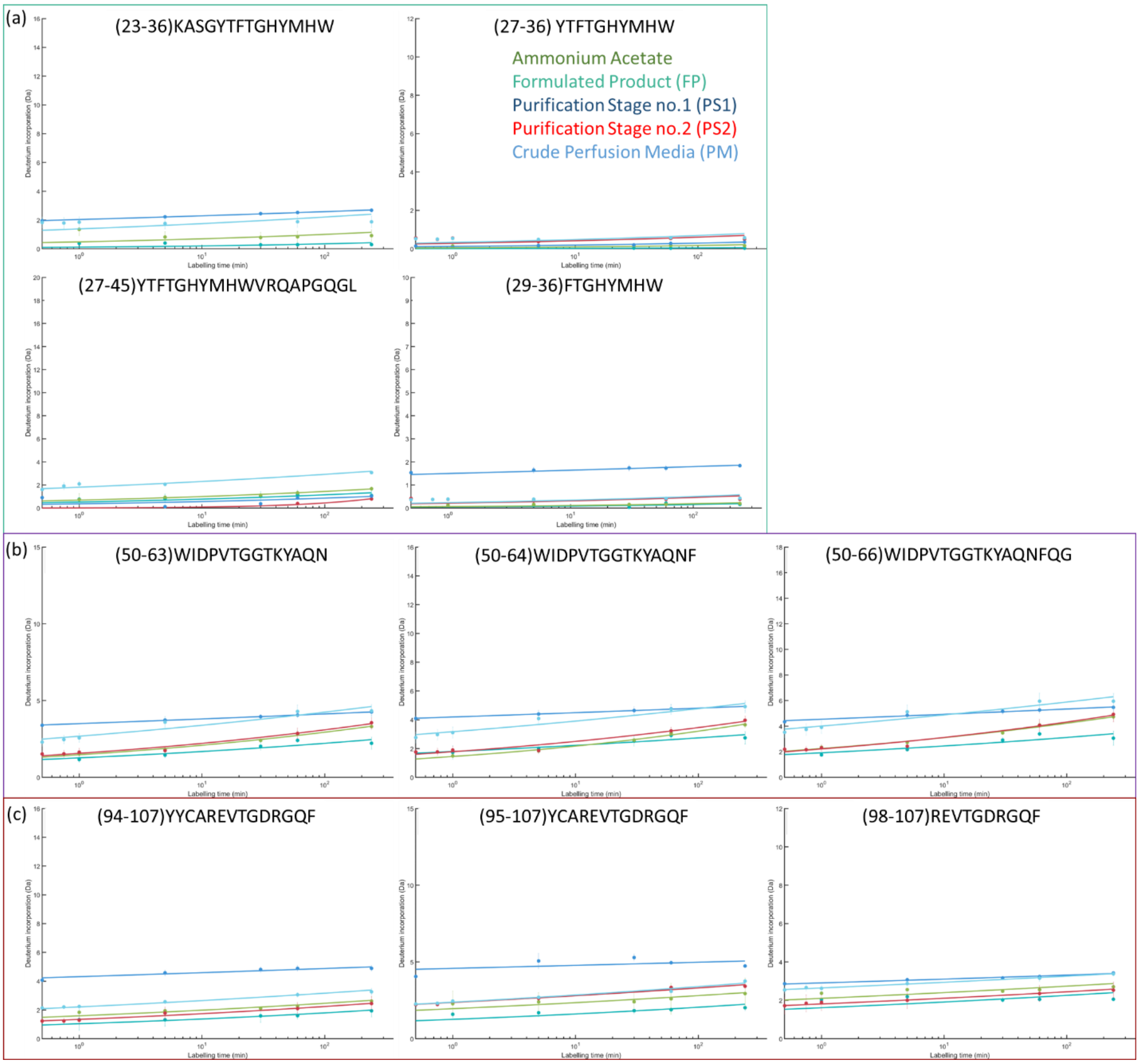

Figure S5: Uptake plots generated using HDflex for heavy chain peptides covering the (a) first complementarity determining region, CDRH1, (b) second, CDRH2, and (c) third CDRH3.

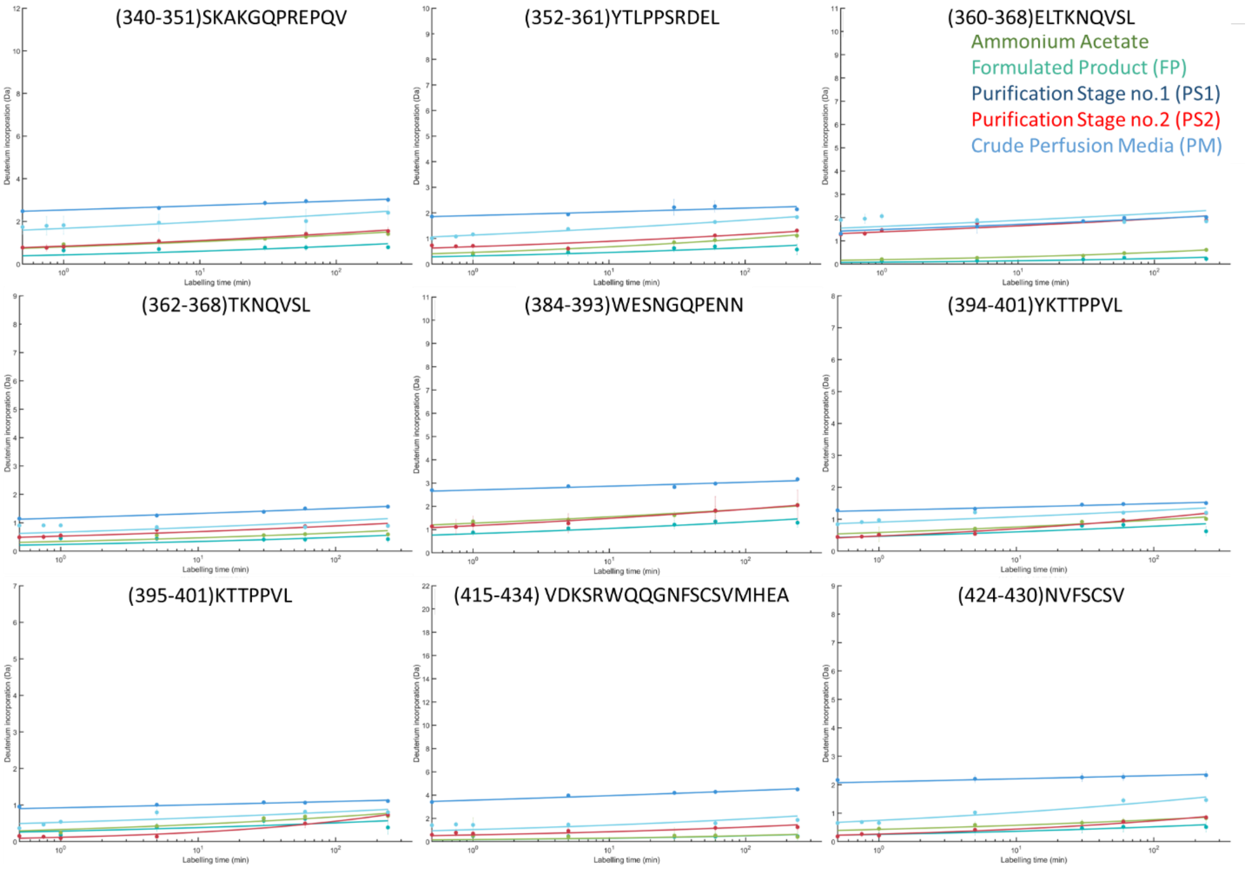

Figure S6: Uptake plots generated using HDflex for heavy chain peptides covering the third constant domain, CH3.

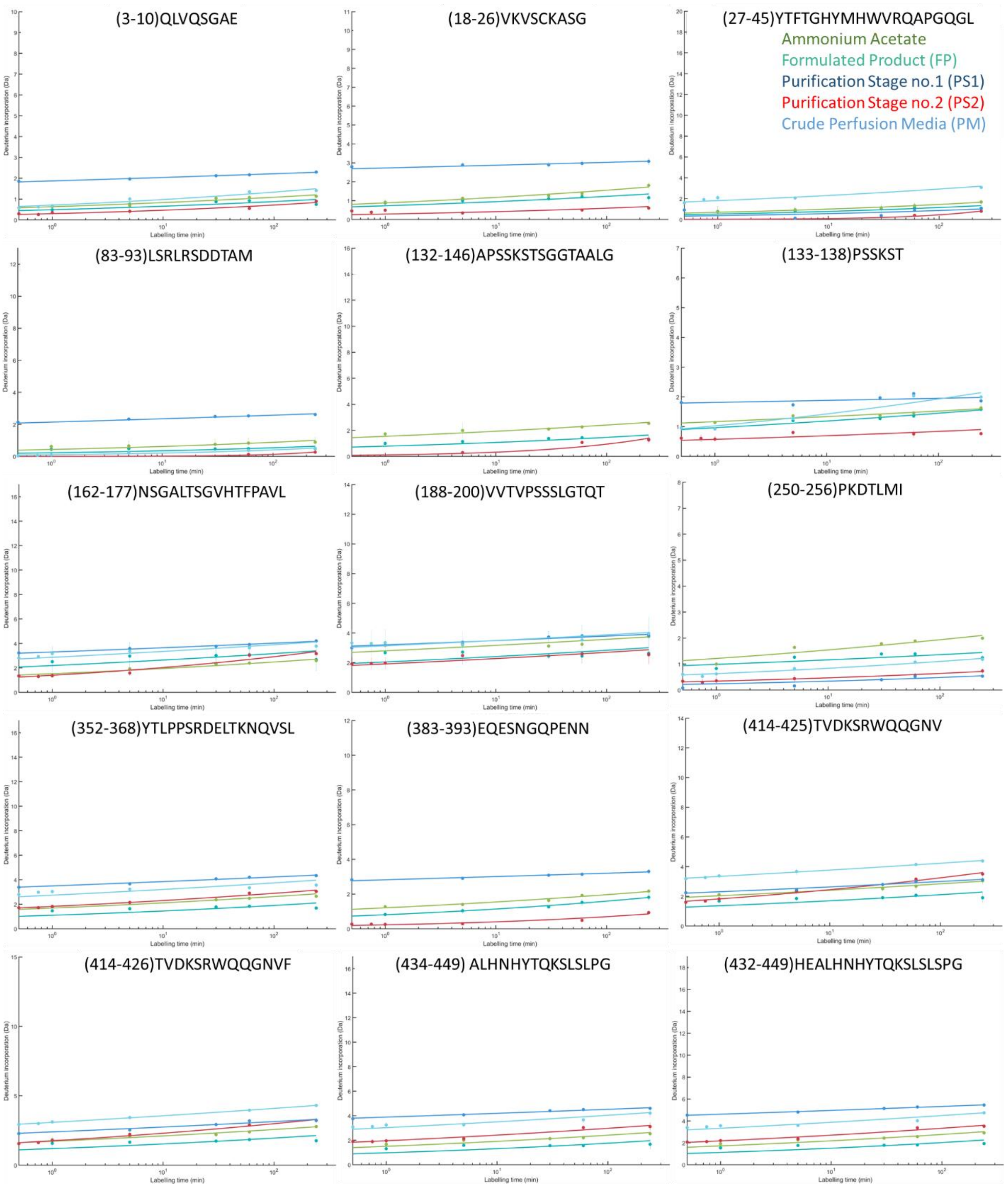

Figure S7: Uptake plots generated using HDflex for heavy chain peptides (VH-CH2) presenting significant uptake in the formulation solution (FP).

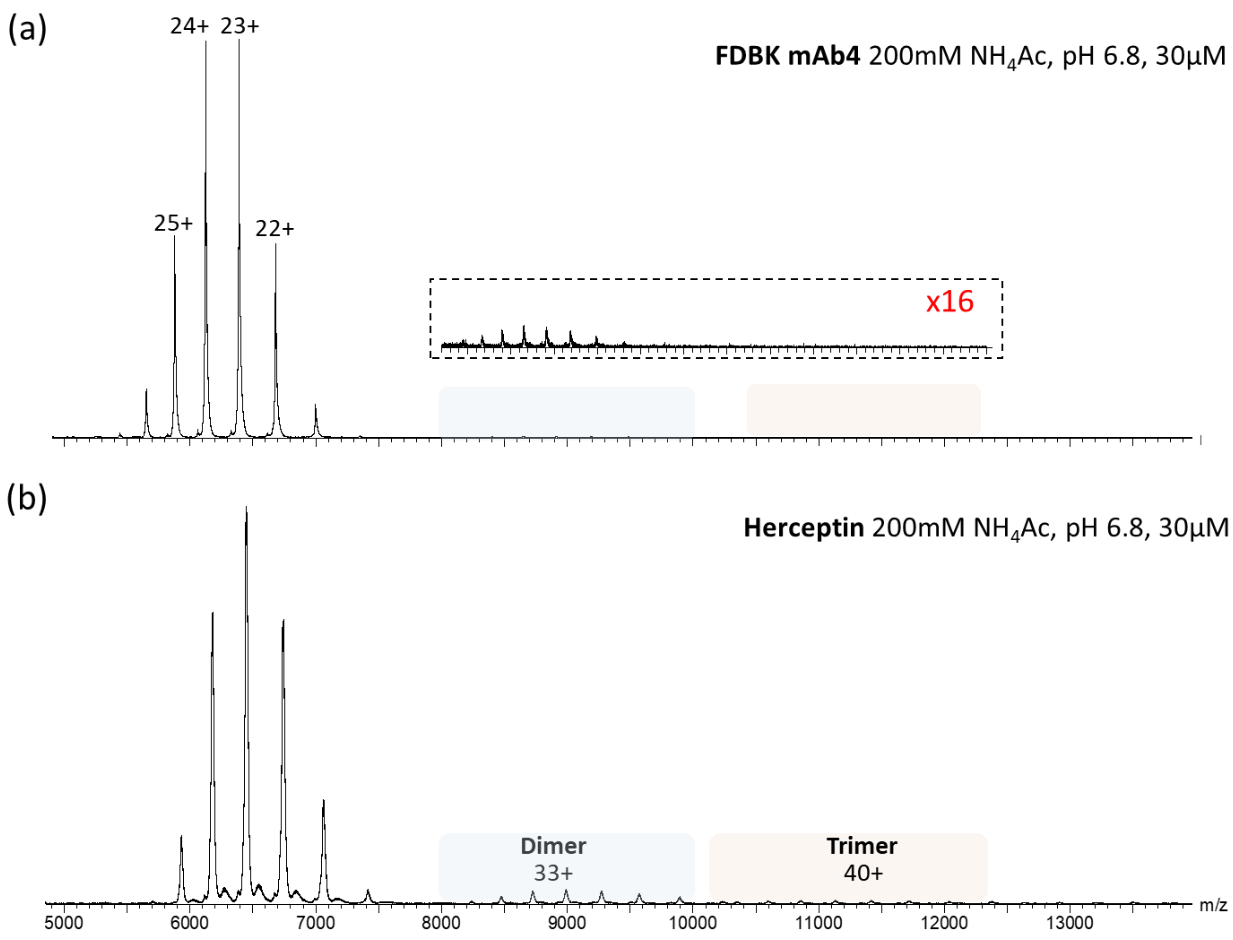

Figure S8: Aggregation propensity studies using Native-MS. FUJIFILM Diosynth Biotechnologies mAb4 (a) and Herceptin mAb (b) were sprayed from similar solution conditions of 200mM NH4Ac, pH 6.8. At 30µM concentration, Herceptin presents higher aggregation propensity compared to mAb4. The inset in (a) is the CSD of the dimer detected after magnification of 16 times in the region.

Although the native and activated IM-MS data were highly reproducible for Herceptin and mAb4 in 200mM NH_4Ac, pH 6.8 the heat map of the mAb4 in 500mM NH_4Ac, pH 6.8 were very different (Figure S9). The main difference between the mass selected 23+ species was better desolvation and higher glycan resolution when harsher ionisation parameters were used. When activated using identical ORIGAMI parameters, detailed in the Material, Methods and Analysis section, but with harsher ionisation parameters the heat map shows trends of a more compact initial conformer with hardly a single transition (T1) (Figure S9b). The mass resolution is compromised under more native-like source conditions (inserts Figure S13). In addition, the initial conformer is bypassing the compaction substage and transitions to the second conformer at lower voltages (48V harsher vs 63.5V softer ionisation). This is helpful to show how at the cost of optimising ion transmission and glycan resolution sometimes we can unintentionally cause ion heating and result in conformers that are not representative of native structures.

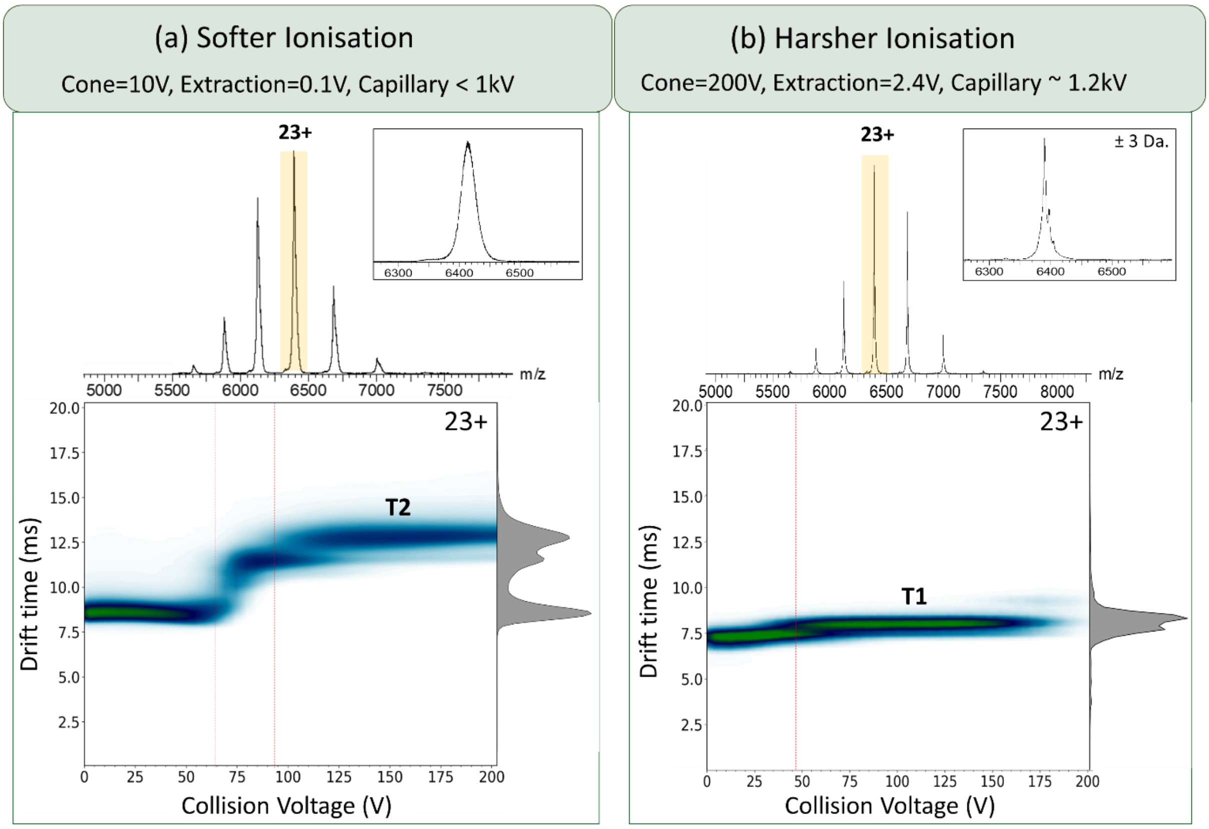

Figure S9: Native mass spectrometry data and activated IM-MS data of mAb4 sprayed from 500mM NH_4Ac, pH 6.8 under soft ionisation parameters (a) and harsher ionisation parameters (b). From the native mass spectrums, a shift to lower charge states is observed while the width of the charge state distribution (CSD) remains the same for both datasets. mAbs present similar charge state envelopes under different ionisation conditions but similar solution conditions. The main difference between the mass selected base peaks is a sodium adduct. Under soft ionisation, mAb4 undergoes two transitions (T1, T2) (a), whereas under harsher ionisation, mAb4 shows trends of a compact conformation with much earlier drift times prior to activation. In addition, only a single transition (T1) was observed with lower activation voltage requirements. At the cost of

better desolvation, resolution and ion transmission the initial conformation resulting in (b) might not be the most native-like.

Enzymatic Digestion for glycan analysis

Following the application note by Genovis AB, the mAb4 sample was diluted with 100 mM Ammonium Acetate, pH 6.0 at 2 mg ml^{-1} (~ 14µM) and IdeS was reconstituted with MilliQ water at (67 units/µL). IdeS and mAb4 were mixed in a 1:1 ratio and left to incubate for 1hr at 37 C. For direct infusion experiments, the sample was buffer-exchanged in 100mM Ammonium Acetate, pH 6.8, and prepared at 0.15 mg ml^{-1} (~ 1µM). MS spectra were obtained to confirm the complete enzymatic digestion. LC-MS experiments were performed on a Vion IMS Q ToF instrument. The samples were prepared at 0.6 mg ml^{-1} (~ 4µM) and the domains were eluted from an Acquity UPLC Protein Column (BEH C4, 300A, 1.7µm, 2.1mm x 100 mm column from WATERS) using an 8-minute gradient and a flowrate of 0.4µL min^{-1}. The aqueous mobile phase was composed of MilliQ water and 0.1% formic acid, and the organic mobile phase of 95% acetonitrile, 5% MilliQ water, and 0.1% formic acid. Data acquisition and analysis were done in Water's UNIFI, and for deconvolution, the MaxEnt1 function was used.

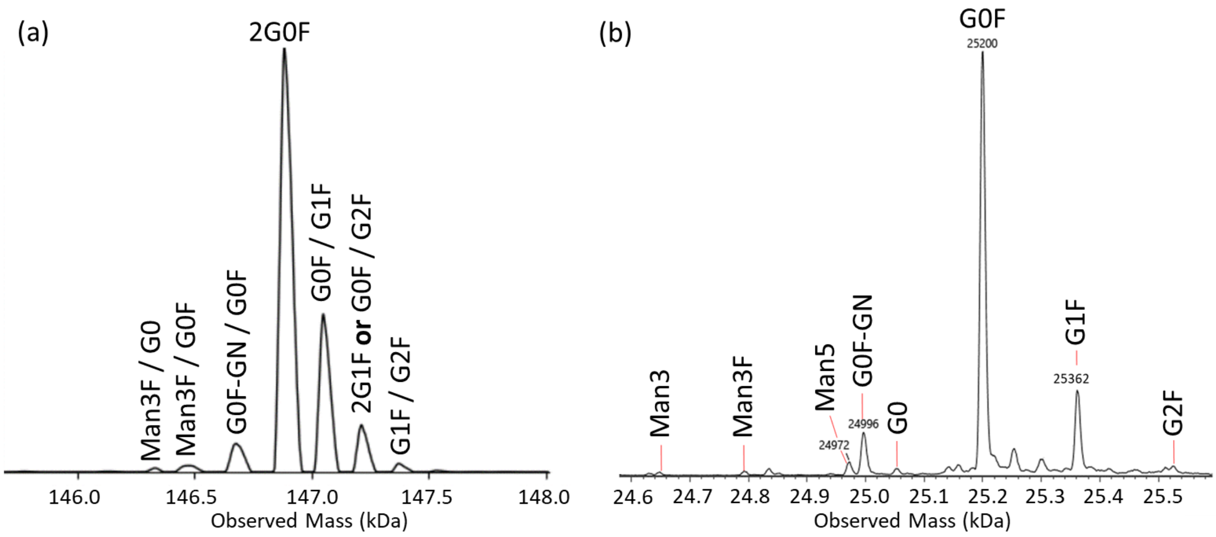

Figure S10: Deconvoluted mass spectrum associated with the glycosylated full-length mAb4 following native MS (a) and Fc/2 antibody subunit following incubation with IdeS (LC-MS). The peaks were assigned based on the comparison between the deconvoluted peaks using UniDec (a), MaxEnt1 (b) and the theoretically calculated values.

Table S1: A summary of the LC-MS results for the digested mAb4. The IgG1 antibody was incubated with IdeS, which cleaves below the hinge, and results in two Fc/2 domains. The relative MS intensity allows for the estimation of the Fc-glycan occupancy.

Glycans	Observed mass (Da)	Expected mass (Da)	Intensity (%)	Mass error (mDa)	Mass error (ppm)
Man3	24647.466	24647.353	0.88	0.113	4.6
Man3F	24793.541	24793.494	7.14	0.047	1.9
Man5	24971.647	24971.634	3.12	0.013	0.5
G0F-GlcNAc	24996.734	24996.686	19.49	0.048	1.9
G0	25053.740	25053.738	1.12	0.003	0.1
G0F	25200.094	25199.879	100	0.214	8.5
G1F	25362.003	25362.019	20.77	-0.017	-0.7
G2F	25524.158	25524.160	0.78	-0.002	-0.1

Table S2: Summary of the native MS results for mAb4. A table for the deconvoluted masses of the glycan pairs detected is shown. The expected mass includes 4 N-terminal conversion of Q to pyroQ isolated to the Fab domain, considering the masses of the Fc/2 domain in Table S1 are of high accuracy while excluding any Q to pyroQ conversions.

Intensity %	Observed Mass (Da.)	Expected Mass (Da.)	Mass Error (Da.)	Glycosylation Pair
0.90	146396.80	146395.94	-0.86	Man3F **/** G0
1.40	146542.56	146542.08	-0.48	Man3F **/** G0F
6.36	146745.17	146745.27	0.11	G0F-GN **/** G0F
100	146948.50	146948.47	-0.57	G0F-G0F
35.19	147113.00	147110.61	-5.26	G0F **/** G1F
8.90	147271.13	147272.75	-4.96	2G1F **or** G0F-G2F
1.59	147437.07	147434.89	-2.18	G1F-G2F **or** G1F-G2F

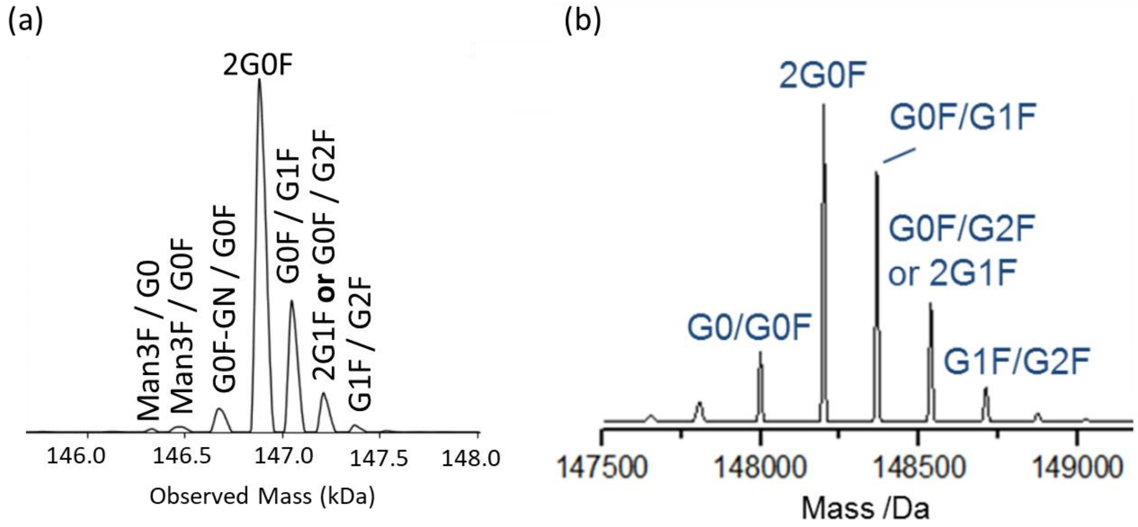

Figure S11: Glycosylation heterogeneity comparison between FUJIFILM Diosynth Biotechnologies mAb4 (a) and Herceptin mAb[34] (b). Although the most abundant glycan pair is 2G0F in both mAb4 and Herceptin, the latter exhibits overall heavier glycosylation, considering the heavier glycan pairs have almost twice the intensity from those detected in mAb4. Reproduced from ref [34]. Copyright 2016 Upton et.al, published under the CC BY 4. 0 License https://creativecommons.org/licenses/by/4.0/deed.en

Table S3: Interaction Parameters, kD, calculated using Dynamic Light Scattering (DLS) as a function of solution condition for mAb4 during purification/formulation and Herceptin in a MS-Compatible solution. PS1 was tested at high ionic strength (200mM) to assess any remaining self-association interactions in solution.

**Sample**	**Composition (mM)**	**pH**	**k_D (ml/g)**	**STDEV**
**Perfusion media (PM)***	**Conditioned with 200 NaCl**	**~8**	**-31.22**	**15.74**
Purification 1 (PS1) eluate	50 mM Na - Acetate	3.5	23.08	1.04
Purification 1 (PS1) eluate	200 mM Na - Acetate	3.5	17.35	0.65
Purification 2 (PS2) eluate	~350 mM Na - Acetate	6	-27.94	3.05
Formulation Product (FP)	20 mM Na-Phosphate, 7.5% w/v Sucrose, 0.01% w/v Polysorbate 20	6	-14.69	1.44
**mAb4** buffer exchanged	200 mM NH_4Ac	6.8	-15.83	0.65
**Herceptin** buffer exchanged	200 mM NH_4Ac	6.8	-23.42	3.59

* The kD calculation for mAb4 in the Perfusion media matrix supplemented with 200mM NaCl, presented a significant STDEV for the three replicates. It is indicative of the protein aggregates in the sample and hence an accurate kD value could not be measured.

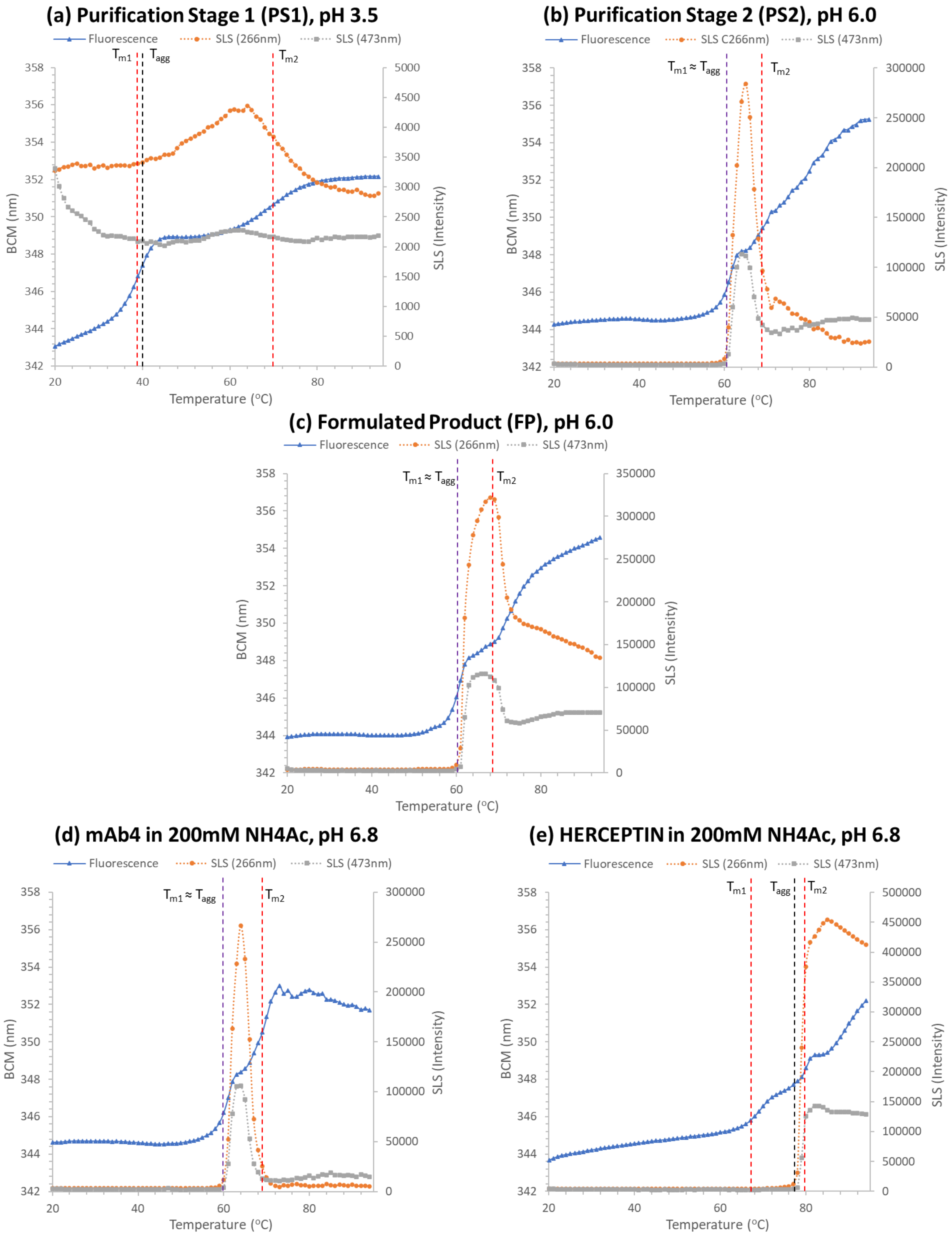

Figure S12: Structural and Colloidal Stability studies using Differential Scanning Fluorimetry (DSF) coupled with Static Light Scattering (SLS). The average fluorescence readings (blue line -Δ-), and SLS intensities for the UV laser-266nm (orange -□-) and blue laser -473nm (grey line -○-) as function of temperature ramping (20-95ºC) for mAb4 in the solutions used for PS1 (a), PS2 (b), and FP (c).The stability of mAb4 and Herceptin was also assessed and compared in an MS-Compatible solution shown in (d) and (e) respectively. Solution conditions are detailed in Table S3. The melting temperature (Tm), and aggregation onset temperature (Tagg) are indicated with the red and black line, respectively. Due to the heterogeneity of the perfusion media solution (PM), high interference was detected mainly in the fluorescence readings but with the SLS as well.

Table S4: Volume and concentration requirements for the mass spectrometry and orthogonal techniques used for the experiments. The low volumes required for native MS and aIM-MS provide continuous spraying for extended periods. The high sensitivity of MS instrumentation results in low sample concentration requirements. In contrast, orthogonal techniques require larger sample volumes compared to the microliter (µL) requirements of MS for triplicate runs.

**Method**	**Protein Concentration (mg/mL)**	**Volume required**
Native MS	0.147 – 1.469<br>(Instrument Dependent)	5 µL per tip
Activate IM-MS	1.469	5 µL per tip
HDX-MS	~ 2.938	5 µL per deuteration point
DLS<br>(concentration gradient)	0.5 – 30	25µL per well
SLS and DSF<br>(UNCLE)	0.05 – 300 mg/mL<br>IgG (Protein Dependent)	8.5 µL per quartz cuvette

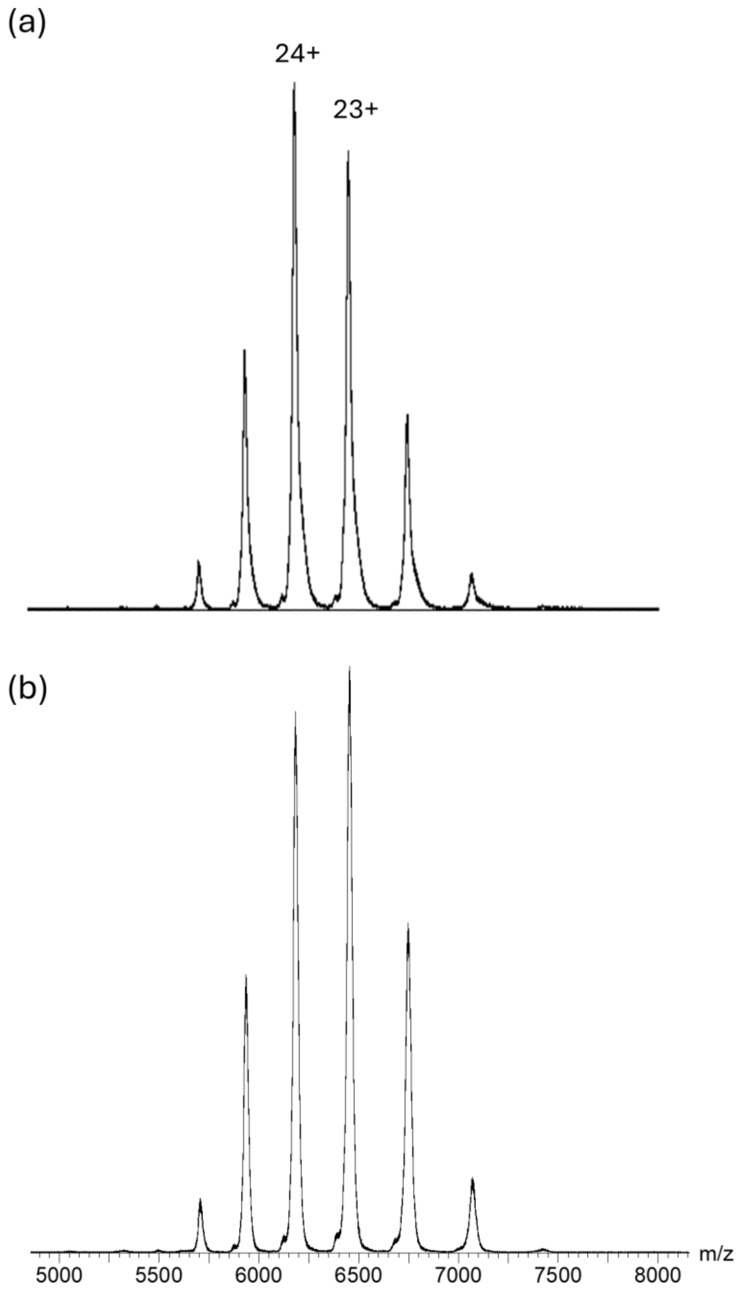

Figure S13: Native mass spectrometry data for (a) 200mM NH_4Ac, and (b)Herceptin in 500mM NH_4Ac. A shift to lower charge states is observed at higher salt concentrations, while the width of the charge state distribution (CSD) remains the same as that at 200mM NH_4Ac.

Ion mobility mass spectrometry on mAb4 and Herceptin

For traveling wave ion mobility separation (TWIMS), the IMS cell was operated at 3.0 mbar with a 40 V wave height and a 500 ms^{-1} wave velocity (Table S5). Each variant was analyzed in triplicate, and results were averaged. Power-law CCS calibration was conducted using concanavalin A (conA) and alcohol dehydrogenase (ADH). Global CCS distributions were obtained by aligning the CCS ranges for individual charge states (6000–9000 Å^2) and summing them. Gaussian fitting was applied to the global distributions to assess the relative proportions of distinct conformational families.

Table S5: Synapt G2Si (TWIMS) tuning parameters used for the IM-MS experiments

**Source voltages (V)**	
Capillary voltage (kV)	0.95 - 1.2
Source temperature (°C)	40
Sampling Cone	50
Source Offset	0
Trap Gas Flow (mL/min)	6
Helium Cell Gas Flow (mL/min)	180
IMS Gas Flow (mL/min)	90
**DC Voltages**	
Trap Collision Energy (V)	5
Trap DC Entrance	3
Trap DC Bias	45
Trap DC	0
Trap DC Exit	0
IMS DC entrance	15
Helium Cell DC	30
Helium Exit	-5
IMS Bias	1.7
IMS DC Exit	1
Transfer Collision Energy (V)	50
Transfer DC entrance	5
Transfer DC exit	15
**Wave Velocities (ms^{-1}), and heights (V)**	
Trap wave velocity	311
Trap wave height	4
IMS wave velocity	500
IMS wave height	40
Transfer wave velocity	380
Transfer wave height	4
**Pressures (mbar)**	
Backing	4.85E+00
Source	2.61E-03
Sample Plate	1.14E+03
Trap	4.07E-02
Helium Cell	4.48E+00
IMS	3.00E+00
Transfer	3.85E-02
TOF	1.32E-06

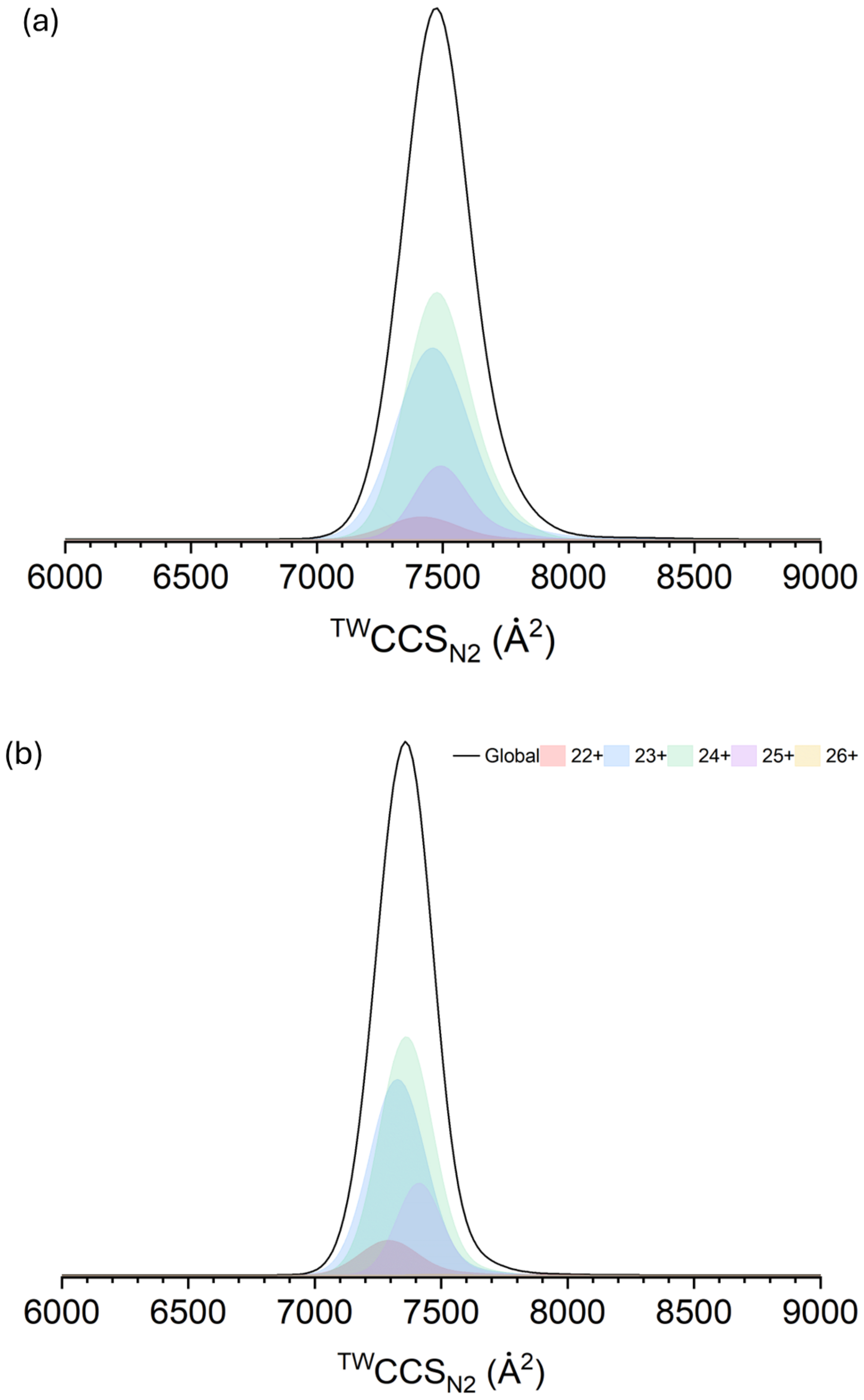

Figure S14: Collision cross section (CCS) distribution for (a) mAb4 and (b) Herceptin. Charge states are color-coded as follows: 22+ (red), 23+ (blue), 24+ (green), 25+ (purple), and 26+ (yellow). The global CCS distribution is shown in a black solid line. Herceptin exhibits narrower CCS distributions across all charge states compared to mAb4. On average, the CCS values for Herceptin are approximately 1nm^2 lower than those of mAb4.

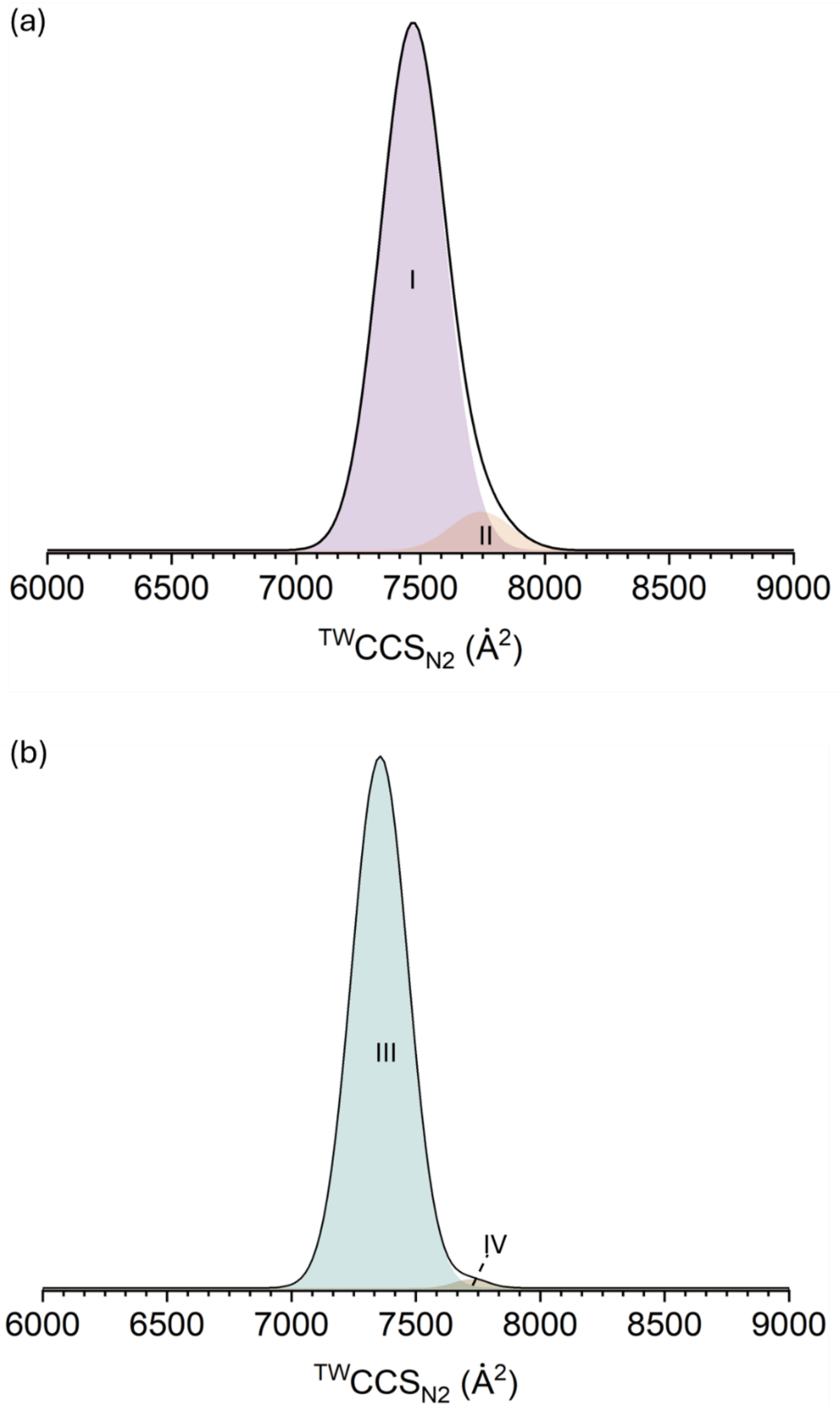

Figure S15: Fitted global collision cross section (CCS) distributions for (a) mAb4, and (b) Herceptin derived by the summing of all charge states observed. Two conformation families I, II for mAb4 (a) and III, IV for Herceptin (b) were found in both antibodies.
